# ERK/CREB and p38 MAPK/MMP14 Signaling Pathway Influences Spermatogenesis through Regulating the Expression of Junctional Proteins in *Eriocheir sinensis* Testis

**DOI:** 10.3390/ijms25137361

**Published:** 2024-07-04

**Authors:** Hong-Yu Qi, Zhan Zhao, Bang-Hong Wei, Zhen-Fang Li, Fu-Qing Tan, Wan-Xi Yang

**Affiliations:** 1 The Sperm Laboratory, College of Life Sciences, Zhejiang University, Hangzhou 310058, China; 12007069@zju.edu.cn (H.-Y.Q.);; 2 School of Medicine, Zhejiang University, Hangzhou 310003, China; drtfq@zju.edu.cn

**Keywords:** MAPK, intercellular junction, hemolymph–testis barrier, spermatogenesis, *Eriocheir sinensis*

## Abstract

The hemolymph–testis barrier (HTB) is a reproduction barrier in Crustacea, guaranteeing the safe and smooth process of spermatogenesis, which is similar to the blood–testis barrier (BTB) in mammals. The MAPK signaling pathway plays an essential role in spermatogenesis and maintenance of the BTB. However, only a few studies have focused on the influence of MAPK on crustacean reproduction. In the present study, we knocked down and inhibited MAPK in *Eriocheir sinensis*. Increased defects in spermatogenesis were observed, concurrently with a damaged HTB. Further research revealed that es-MMP14 functions downstream of ERK and p38 MAPK and degrades junctional proteins (Pinin and ZO-1); es-CREB functions in the ERK cascade as a transcription factor of ZO-1. In addition, when es-MMP14 and es-CREB were deleted, the defects in HTB and spermatogenesis aligned with abnormalities in the MAPK. However, JNK impacts the integrity of the HTB by changing the distribution of intercellular junctions. In summary, the MAPK signaling pathway maintains HTB integrity and spermatogenesis through es-MMP14 and es-CREB, which provides insights into the evolution of gene function during barrier evolution.

## 1. Introduction

The successful process of spermatogenesis is critical for male reproduction and occurs in the testes. In mammals, some spermatogonia maintain their number through mitosis to support further spermatogenesis, while others divide into primary spermatocytes through the first meiosis. During this process, preleptotene spermatocytes traverse the blood–testis barrier (BTB), which relies on adjacent Sertoli cells. These cells enter the apical compartment from the basal compartment and develop into haploid early sperm cells. Subsequently, germ cells continue to progress and are released into the seminiferous tubules after spermiogenesis [1,2]. Sertoli cells are the only somatic cells in the seminiferous epithelium; they not only provide nutrition for the germ cells but also offer a rampart-like structure support, the BTB [3]. Spermatogenesis requires an intact and functional BTB [4], and the meiosis of spermatocytes is disrupted when the BTB is damaged [5].

The BTB consists of several types of junctions between adjacent Sertoli cells: tight junctions (TJs), basal ectoplasmic specialization (basal ES), desmosomes and gap junctions (GJs) [6,7]. Accurate localization between Sertoli cells is essential for the formation of a functional BTB. However, to facilitate the crossing of spermatocytes through the BTB, it is sometimes necessary to coordinate the reorganization of these proteins [8]. It has been reported that abnormalities in the biosynthesis, degradation, distribution, endocytosis and recycling of junction proteins in seminiferous tubules destroy the stability of the BTB and subsequently impact spermatogenesis [9]. The absence of claudin-11 (a type of TJ in the BTB) results in impaired spermatogenesis in mice, with development halting at the spermatocyte stage [10]. The transcription process plays a role in regulating the expression of the junction proteins. ERK1/2, which phosphorylates CREB (cAMP response element binding protein) and ATF-1, transcription factors that cause the upregulation of claudin-1 and claudin-5, enhance TJ formation [11]. In addition, in the nonclassical testosterone signaling pathway, ERK1/2 also actives CREB and triggers the expression of ZO-1 and claudin-5 [12]. These factors impact the integrity of the BTB in terms of protein biosynthesis. In addition, another reason for protein expression is degradation. Matrix metalloproteases (MMPs) are reportedly involved in junction dynamics, and they degrade the components of the basement membrane in seminiferous tubules, thereby regulating the steady state of junctional proteins (such as occludin) in the BTB [13]. In primary cultured Sertoli cells, the secretion of MMP9 increases TJ degradation, which is related to the activity of ERK1/2 [14]. In addition to its effects on occludin, MMPs also impact the expression level of ZO-1 in other barriers. This effect can be partially counteracted by MMP inhibitors [15]. MMP2 reduces the expression of occludin and causes a gap between adjacent Sertoli cells, and the levels of laminin-γ3 and β1 are also decreased; these detrimental effects can be alleviated by treatment with an MMP2 inhibitor [16]. Therefore, transcription factors and MMPs regulate the level of junctional proteins in the BTB and guarantee the smooth progression of spermatogenesis.

Multiple signaling pathways are associated with spermatogenesis, such as the PI3K/AKT pathway [17], mTOR pathway [18], testosterone signaling pathway [19], MAPK [20] and so on. The MAPK signaling pathway includes four main branch pathways, namely, the ERK1/2, JNK, p38 MAPK and ERK5 pathways, which operate in both germ cells and Sertoli cells and participate in the production of male gametes [21,22]. Moreover, the MAPK signaling pathway has been shown to be a critical factor in maintaining the integrity and function of the BTB. Certain heavy metals [23,24,25], environmental pollutants [26,27,28] and some hormones [29] induce BTB disruption through MAPK. Indeed, during spermiation, the ES needs to be disassembled, a process that coincides with an increase in ERK activity [30], and it seems that the activity of ERK accelerates junction disintegration. However, the follicle-stimulating hormone (FSH) facilitates the formation of TJs in Sertoli cells by activating ERK [31,32], and ERK phosphorylates CREB to promote the expression of ZO-1 and claudins in the nonclassical testosterone signaling pathway [12]. Depending on the features of the upstream stimulus, JNK can either promote or prevent the stabilization of junctions in the BTB. For instance, under the influence of the paracrine factor TNF-α, JNK accelerates MMP transcription and perturbs the BTB [33], but the activity of JNK alleviates the cadmium-mediated destruction of the BTB [34]. It has been reported that upon stimulation by hexafluoropropylene oxide, p38 MAPK disrupts BTB integrity and enhances the degradation of occludin through MMP9 [35]. Additionally, Miao et al. [36] suggested that p38 MAPK directly affects the expression of junction proteins in the BTB, and they substantiated this conclusion in vivo and in vitro. Another RNA-seq study revealed that p38 MAPK might be related to the functional BTB through regulating actin and the microtubule cytoskeleton [23].

Although the MAPK signaling pathway has been widely studied in the mammalian BTB, this finding remains elusive in aquatic species. *Eriocheir sinensis* is an important economic aquatic species in China, and research on its molecular mechanism of spermatogenesis is beneficial for improving the quality of male gametes, guaranteeing higher yields and economic value for crab farming. The events of spermatogenesis in *E. sinensis* are similar to those in mammals; however, with the unique characteristic of producing nonflagellar sperm, *E. sinensis* is regarded as a modal species for understanding spermatogenesis in Crustacea. In previous studies, key factors in the MAPK signaling pathway, including ERK, JNK and p38 MAPK, were identified and characterized. While JNK is involved in antimicrobial function and the innate immune response [37,38], ERK and p38 MAPK are highly expressed in the testis of *E. sinensis* and play crucial roles in the acrosome reaction [39,40]. In addition, in our previous studies, we constructed a modal for a novel barrier structure in the testis of *E. sinensis*, known as the hemolymph–testis barrier (HTB), which shares a similar position and function to the BTB observed in mammals [41]. Next, we discovered that the mTOR and Wnt/β-catenin signaling pathways were related to the integrity of HTB, and we detected that the deletion of the motor protein myosin VI led to disruptions in HTB integrity and function, accompanied by alterations in the MAPK signaling pathway. However, the specific mechanisms through which the MAPK signaling pathway influences spermatogenesis and HTB integrity remain unknown and require further investigation.

Here, we employed dsRNA and the specific inhibitors U0126, SP600125 and SB203580 to interfere with or inhibit ERK, JNK and p38 MAPK, respectively, in the testis of *E. sinensis*. By conducting an HTB integrity assay, using Western blotting and immunofluorescence to evaluate the expression and distribution of cell junction proteins, we found that the deletion of ERK or p38 MAPK destroyed the HTB by influencing the expression of junctional proteins, whereas the deficiency of JNK disturbed the HTB by changing the localization of junctional proteins. To explore the further reasons for these changes in cell junctions, we identified two kinds of es-MMPs and a transcription factor, es-CREB. Furthermore, our research revealed that ERK and p38 MAPK regulated the contents of Pinin and ZO-1 through the degradation function of es-MMP14; meanwhile, es-CREB modulated the transcription of ZO-1 as a transcription factor downstream of ERK. To further support our conclusions, we overexpressed both MAPK and inactive MAPK factors in vitro and then examined the changes in the expression of cell junction proteins, MMPs and CREB. We obtained results similar to those of the in vivo experiments. Briefly, our findings provide the first insights into the influence of the MAPK signaling pathway on intercellular junctions in Crustacea and illustrate the important molecular mechanisms by which this pathway maintains HTB integrity and facilitates spermatogenesis.

## 2. Results

### 2.1. MAPK Deficiency or Inhibition Results in Abnormal Spermatogenesis in E. sinensis

**Figure 1:**
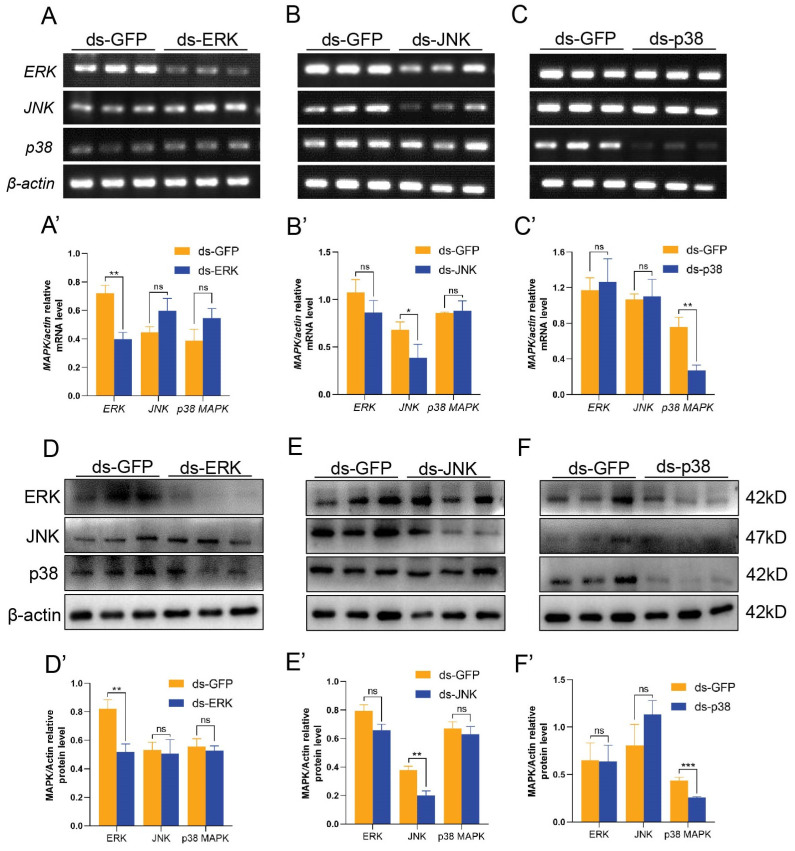
The efficiency of knockdown after injection of dsRNA against ERK, JNK and p38 MAPK in vivo. (**A**–**C**) sqPCR results showing the mRNA levels of *ERK*, *JNK* and *p38 MAPK* after knockdown. (**A′**–**C′**) Analysis of the mRNA level of *ERK*, *JNK* and *p38 MAPK* in the interference groups by ImageJ2 v2.15.0. (**D**–**F**) Western blotting results showing the protein levels of ERK, JNK and p38 MAPK in the knockdown groups. (**D′**–**F′**) Analysis of protein levels by ImageJ2 after MAPK signaling pathway knockdown. * *p* < 0.05 indicates a significant difference between two groups; ** *p* < 0.01 indicates an extremely significant difference; *** *p* < 0.001 indicate an extremely significant difference. ‘ns’ indicates that there was no significant change.

To investigate the specific function of the MAPK signaling pathway in spermatogenesis in *E. sinensis*, we silenced ERK, JNK and p38 MAPK. dsRNA was designed to disturb the transcription level of MAPK, and dsRNA against GFP was used as a control. sqPCR and Western blotting were used to detect the efficiency of the knockdown, revealing that ERK, JNK and p38 MAPK were significantly lower at both the mRNA and protein levels in the knockdown group, which satisfied further studies (Figure 1A–F). In order to determine the role of ERK, JNK and p38 MAPK, we checked the specificity of each group. The sqRNA and Western blotting results showed that the interference of one gene did not affect the other two MAPK genes (Figure 1A–F).

**Figure 2:**
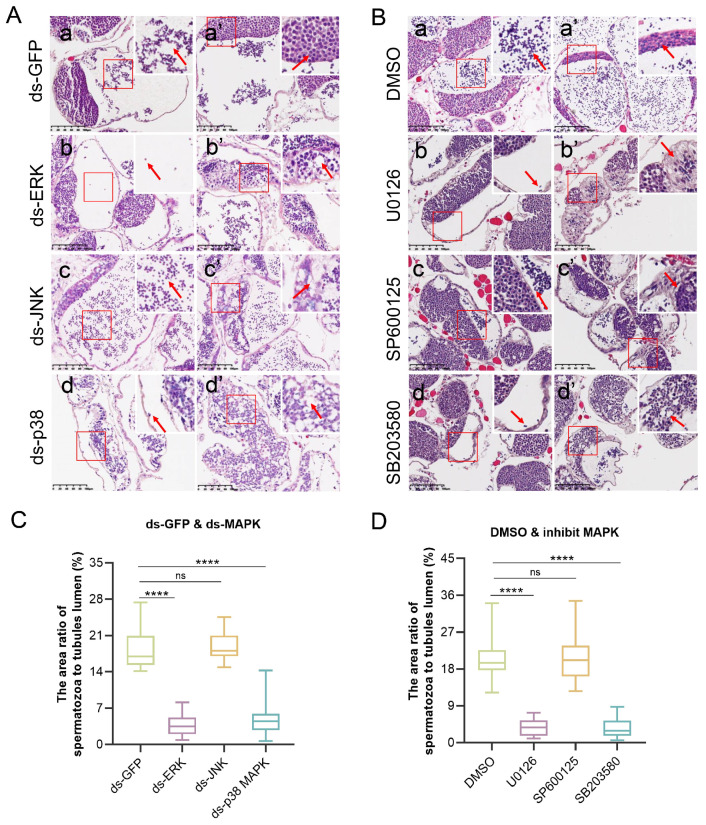
The morphology of testes after interfering or inhibiting ERK, JNK and p38 MAPK in vivo. (**A**) H&E staining results showing interference of the MAPK signaling pathway; (**a**,**c**) enrichment of spermatozoa in the control and dsJNK groups (red arrows pointed to the mature sperm); (**b**,**d**) loss of mainly mature sperm (red arrows pointed to the few mature sperm); (**a′**) regular germ cells in the control group (red arrows pointed to the early germ cells in germinal zones); and (**b′**–**d′**) irregular germinal zone in the dsMAPK groups (red arrows pointed to the early germ cells in germinal zones). (**B**) H&E staining results showing the inhibition of MAPK signaling pathway; (**a**,**c**) spermatozoa enriched in the DMSO and SP600125 groups (red arrows pointed to the mature sperm); (**b**,**d**) lost mainly mature sperm (red arrows pointed to the few mature sperm); (**a′**) regular germ cells in the control group (red arrows pointed to the early germ cells in germinal zones); and (**b′**–**d′**) irregular germinal zones in the inhibit-MAPK groups (red arrows pointed to the early germ cells in germinal zones). (**C**,**D**) The area ratio of mature sperm to the evacuation zone of (**A**) and (**B**), respectively. The scale bars are 100 μm in (**A**,**B**). **** *p* < 0.0001 indicate an extremely significant difference. ‘ns’ indicates that there was no significant change.

Since the activity of MAPK is more important for function in addition to its expression level, we treated crabs with inhibitors of ERK (U0126), JNK (SP600125) and p38 MAPK (SB203680), respectively, while DMSO was used as a control. Next, H&E staining revealed significant differences in the morphology of the seminiferous tubules between the control and treatment groups. As we described previously, germ cells were distributed regularly in the control group, early spermatocytes and spermatids were in the germinal zone, and mature spermatozoa were enriched in the evacuation zone (Figure 2(Aa,Aa′,Ba,Ba′)). Surprisingly, many empty lumens were detected in the dsRNA and inhibitor testes, and the early germ cells in the germinal zone were irregular, with increased vacuolization (Figure 2(Ab–Ad′,Bb–Bd′)), which indicates that spermatogenesis was abolished in the knockdown and inhibition groups. To quantify the difference in spermatozoa between the treatment and control groups, we analyzed the area ratio of spermatozoa in the evacuation zone to that in the total evacuation zone from one lumen. Compared with that in the dsGFP group, the percentages in the dsERK and dsp38 MAPK groups were decreased significantly, from 18.2 to 3.9 and 5.1%, respectively (Figure 2C). Similarly, in alignment with the knockdown group, the percentages in the U0126 (3.7%) and SB203580 (3.6%) groups were also lower than in the DMSO (20.5%) group (Figure 2D). This finding further suggested that ERK and p38 MAPK are crucial for spermatogenesis in *E. sinensis* and that abnormal ERK and p38 MAPK function leads to a chaotic position of germ cells and impedes the production of mature and healthy sperm. However, neither the deletion nor the inhibition of JNK changed the number of spermatozoa; these effects just perturbed the germinal zone but did not influence spermatozoa (Figure 2D). Therefore, ERK and p38 MAPK are more important for the normal production of male gametes in *E. sinensis* testes.

### 2.2. The Integrity of HTB in E. sinensis Is Impaired When the MAPK Signaling Pathway Is Deleted or Inhibited

**Figure 3:**
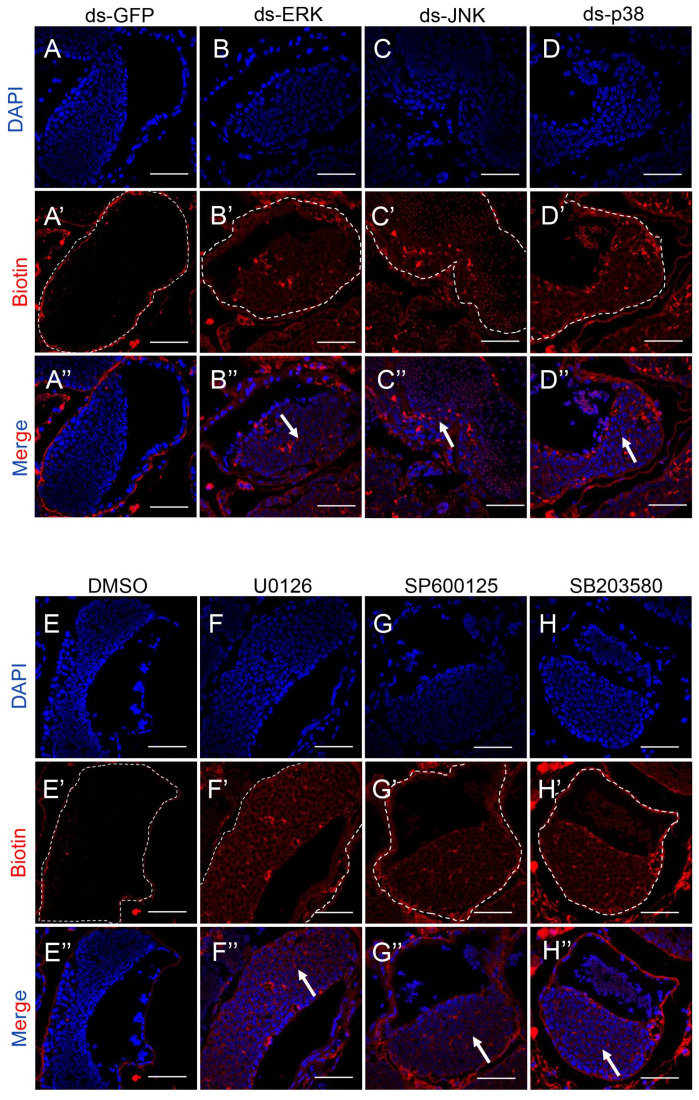
HTB integrity assay in the testis after knocking down or inhibiting ERK, JNK and p38 MAPK in *E. sinensis*. (**A**–**D**) The HTB integrity assay results of the MAPK knockdown groups compared with dsGFP group. (**A**–**A″**) HTB in dsGFP crabs, the red signal of biotin stopped out of the basement of the lumen. (**B**–**D**) HTB in dsMAPK crabs; biotin permeated into tubules in a disordered manner. (**E**–**H**) Results of the HTB integrity assay of the MAPK inhibition groups compared with the DMSO group. In (**E**–**E″**), biotin (red signal) was just located immediately outside the lumen in the DMSO treatment group, but in (**F**–**H**)**,** a mass of biotin flocked in the seminiferous tubules in the U0126, SP600125 and SB203580 groups. The white dotted lines in (**A′**–**H′**) showed the boundaries of seminiferous lumens, and the white arrows in (**B″**–**D″**) and (**F″**–**H″**) pointed to the permeated biotin signals in seminiferous lumens. The bars represent 50 μm.

In our previous study, the deficiency of the motor protein es-Myosin VI disrupted the integrity of HTB and influenced the production of mature sperm, concurrently altering the MAPK signaling pathway [42]. In addition, increased vacuolization in the germinal zone was also a symbol of a poor barrier [43]. Therefore, we checked whether HTB was disrupted by the deletion or inhibition of the MAPK pathway. We carried out the barrier integrity assay as previously described by using biotin-streptavidin immunofluorescence [42]. We found that nearly all the biotin was resisted out of the tubules, and the bright-red signals were located at the base membrane in the dsGFP and DMSO groups, revealing an intact barrier structure (Figure 3A,E). Conversely, substantial biotin signals were observed within the seminiferous tubules in the other knockdown or inhibition groups, which showed that biotin widely permeated into the tubules and intercellular spaces because of the damaged HTB (Figure 3B–D,F–H). In brief, the MAPK signaling pathway is important for the integrity of HTB, even though we did not find significantly reduced spermatozoa after knocking down or inhibiting JNK.

### 2.3. ERK and p38 MAPK Regulate the Expression Levels of Cell Junction Proteins

**Figure 4:**
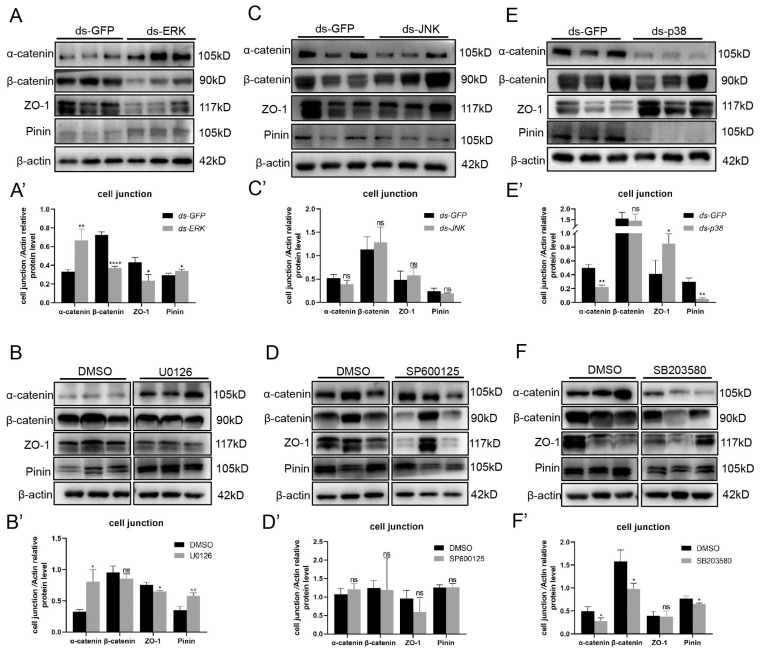
Changes in cell junction protein expression levels after knocking down or inhibiting the MAPK signaling pathway in *E. sinensis* testes. (**A**,**A′**) Western blotting results of α-catenin, β-catenin, ZO-1 and Pinin after injection with dsRNA against ERK compared with those after injection with dsRNA against GFP, and the data were analyzed by ImageJ2 and are showed in (**A′**). (**B**,**B′**) The expression level of junctional proteins after inhibiting ERK by U0126. (**B′**) shows the results analyzed by ImageJ2. (**C**,**C′**) The expression level of junctional proteins in the dsJNK group. The data were analyzed by ImageJ2, as shown in (**C′**). (**D**,**D′**) Western blotting and analysis of the results in the SP600125 group showed changes in junction protein levels compared with those in the DMSO group. (**E**,**F**) The expression levels of four kinds of cell junction proteins in the dsp38 MAPK and SB203580 groups compared with those in the dsGFP and DMSO groups. (**E′**,**F′**) are the results obtained via ImageJ2. * *p* < 0.05 indicates a significant difference between two groups; ** *p* < 0.01 indicates an extremely significant difference; **** *p* < 0.0001 indicate an extremely significant difference. ‘ns’ indicates that there was no significant change.

**Figure 5:**
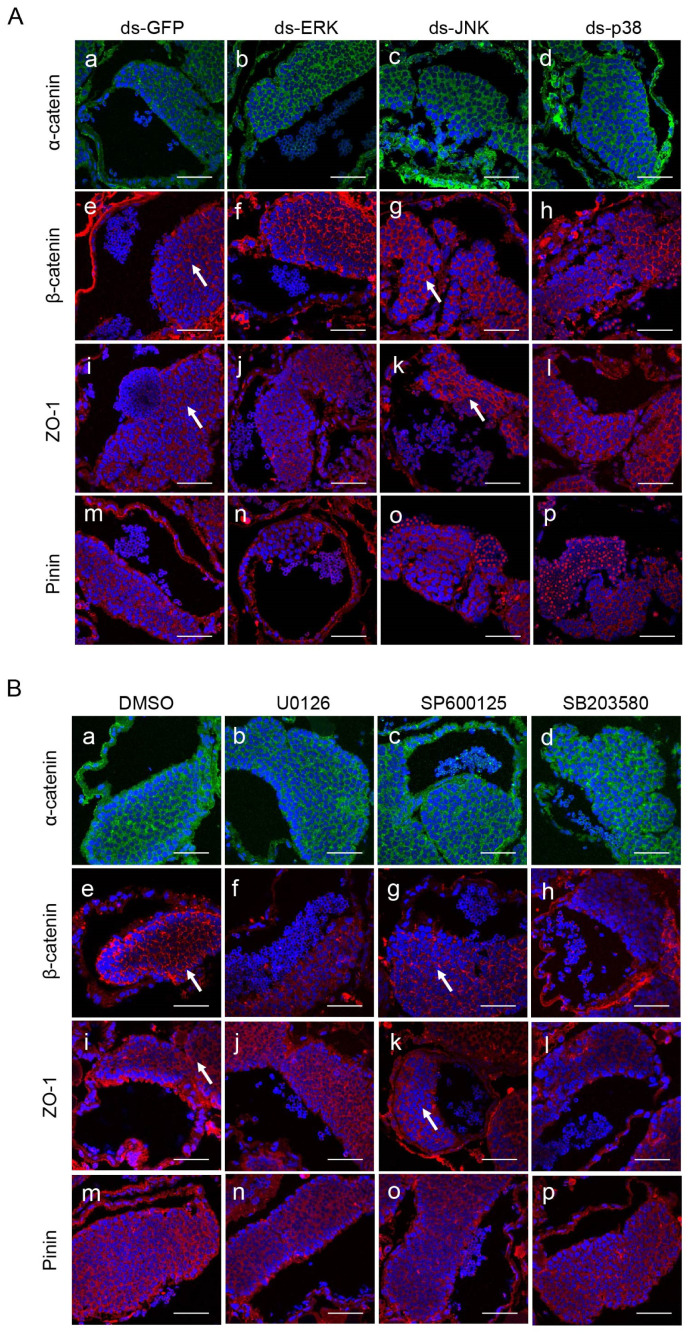
The distribution of α-catenin, β-catenin, ZO-1 and Pinin between intercellular spaces in the MAPK knockdown or inhibition groups in vivo. (**A**) The distribution of junction proteins in the MAPK knockdown groups. (**a**–**d**) Localization of α-catenin (green signal); (**e**–**h**) Localization of β-catenin (red signal); (**i**–**l**) localization of ZO-1 (red signal); (**m**–**p**) localization of Pinin (red signal). The white arrows pointed a wider and brighter fluorescent signal of β-catenin and ZO-1 around nucleus in the dsJNK group, but not in the dsGFP group. (**B**) The distribution of junction proteins in the MAPK inhibition groups. (**a**–**d**) Localization of α-catenin (green signal); (**e**–**h**) localization of β-catenin (red signal); (**i**–**l**) localization of ZO-1 (red signal); (**m**–**p**) localization of Pinin (red signal). The white arrows pointed to a weaker and thinner signal of β-catenin and ZO-1 between early germ cells in the SP600125 group than in the DMSO group. The deficiency or inhibition of ERK or p38 MAPK did not influence the distribution of cell junctions, but after knockdown or inhibition of JNK, the localization of β-catenin and ZO-1 was changed The bars represent 50 μm.

Intercellular junctions are the critical components of the barrier, and we examined four junction proteins in the HTB: the tight-junction protein ZO-1, basal ES proteins α-catenin and β-catenin, and the desmosome protein Pinin. First, we detected the expression levels of these genes through Western blotting and obtained similar results for each pair of interference and inhibition groups. After knocking down or inhibiting the activity of ERK, the expression of α-catenin and Pinin increased dramatically, while that of β-catenin and ZO-1 decreased (Figure 4A,B). There were apparent decreases in the expression of α-catenin and Pinin in the p38 MAPK deficiency and SB203580 treatment groups; nevertheless, ZO-1 was upregulated dramatically (Figure 4E,F). However, the expression of all four junction proteins did not change when JNK was deleted or inhibited (Figure 4C,D). Next, we used immunofluorescence to test the distribution of these intercellular proteins, and both the normal expression level and correct localization indicated intact and functional HTB. According to the results, we did not observe a decreased distribution of junction proteins in ERK or p38 MAPK knockdown or inhibited crabs, and all the proteins were located in the intercellular space in a similar pattern (Figure 5A,B). However, in the JNK knockdown group, a wider and brighter fluorescent signal of β-catenin and ZO-1 appeared around the nucleus, while these signals were weaker and thinner between early germ cells in the SP600125 group than in the DMSO group (Figure 5A,B). In conclusion, the MAPK signaling pathway dysfunctions HTB by regulating intercellular junction proteins in *E. sinensis*. ERK and p38 MAPK disturb HTB by changing protein expression levels, while JNK influences protein distribution in the testis.

### 2.4. Identification and Characterization of es-MMP and es-CREB in E. sinensis Testes

Furthermore, we wanted to explore the downstream mechanisms between the MAPK signaling pathway and junctional proteins. We focused on two kinds of MMPs (es-MMP14 and es-MMP21) and a transcription factor (es-CREB). Uncharacterized es-MMP21 and es-CREB were first cloned and identified.

The CDSs of *es-MMP21* and *es-CREB* (GenBank accession numbers: PP493013 and PP493012) are 2856 bp and 1062 bp, respectively, and they encode 951 aa and 353 aa, respectively (Supplementary Figure S2). We used the online website tool https://web.expasy.org/compute_pi/ (accessed on 1 August 2023) to predict the molecular weights of 107.92 kDa and 35.99 kDa. The multiple sequence alignment results of es-MMP14 and es-MMP21 are shown in Supplementary Figure S3. The online tool SMART (http://smart.embl-heidelberg.de/, accessed on 11 August 2023) was used to predict the secondary structure of es-MMP21 and es-CREB; es-MMP21 had a conserved ZnMc domain from 542 aa to 705 aa, and es-CREB had a conserved BRLZ domain from 294 aa to 351 aa (Supplementary Figure S2). We used MEGA 5.0 (Mega Limited, Auckland, New Zealand) to construct the phylogenetic tree with the NJ method. Es-MMP14 significantly differed from es-MMP21 and did not cluster together; however, they clustered with the same gene of aquatic animals (Supplementary Figure S2).

### 2.5. ERK and p38 MAPK Change the Expression Levels of Pinin and ZO-1 through es-MMP14 but Not es-MMP21 in the Testis of E. sinensis

Previous research in mammals has shown that the MMP is regulated by the MAPK signaling pathway and degrades some tight-junction proteins, influencing their expression level and maintaining barrier integrity; therefore, we identified the MMP in the testis of *E. sinensis*. There are two kinds of MMPs in crabs, es-MMP14 and es-MMP21. In another previous study, Li et al. [44] cloned and characterized es-MMP14 in *E. sinensis*; in the present study, we identified another MMP in this protein family, es-MMP21. Most of the MMPs function in the cell membrane for protein degradation in mammals. To explain their degradation functions, we performed immunofluorescence to observe the localization of es-MMP14 and es-MMP21 in the testis. By costaining with the membrane tracer DIO, we found that es-MMP14 and es-MMP21 both colocalized with the cell membrane in spermatocytes and spermatids (Supplementary Figure S1), suggesting that they could also degrade junctional proteins in the cell membrane.

**Figure 6:**
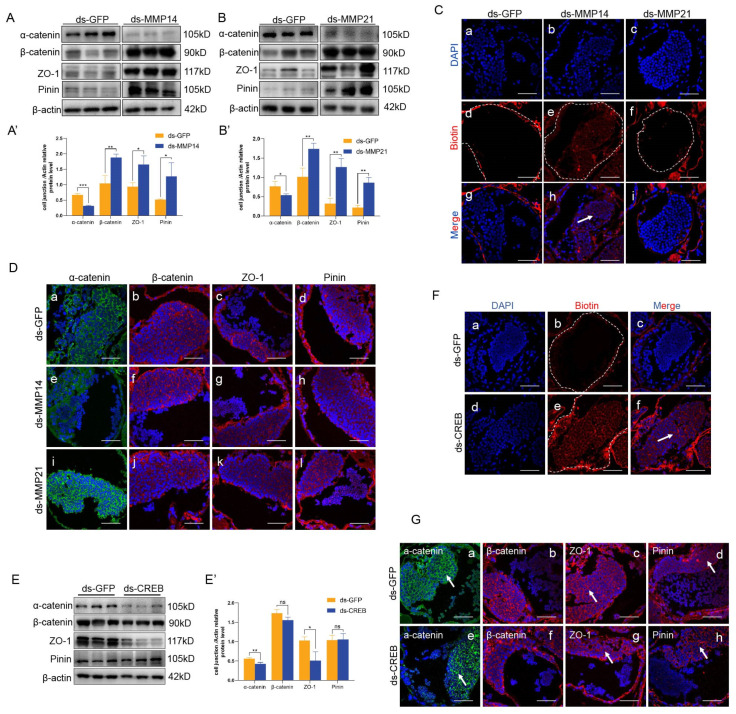
The integrity of HTB after knockdown of es-MMP14, es-MMP21 or es-CREB in *E. sinensis*. (**A**) The expression levels of α-catenin, β-catenin, ZO-1 and Pinin after injection of dsRNA against es-MMP14. (**A′**) The results of (**A**) were examined by ImageJ2. (**B**) The expression levels of cell junction proteins after injection of dsRNA against with es-MMP21. (**B′**) The results of (**B**) were analyzed by ImageJ2. β-catenin, ZO-1 and Pinin were significantly increased in the dsMMP14 and dsMMP21 groups. (**C**) HTB integrity assay results in the dsMMP14 and dsMMP21 groups compared with that in the dsGFP group. HTB was intact in (**a**,**c**,**d**,**f**,**g**,**i**) but damaged in (**b**,**e**,**h**), and biotin (red signal) was detected as a red signal. (**D**) Changes in the localization of junctional proteins after interfering es-MMP14 or MMP21, respectively. The green signals in (**a**,**e**,**i**) represent α-catenin; and the red signals in (**b**,**f**,**j**) were β-catenin, the red signals in (**c**,**g**,**k**) were ZO-1, the red signals in (**d**,**h**,**l**) represent Pinin. They were all distributed among the intercellular spaces. (**E**) Expression levels of junctional proteins in the es-CREB knockdown group. (**E′**) The data of (**E**) were examined by ImageJ2. α-catenin and ZO-1 were dramatically decreased after the knockdown of es-CREB. (**F**) HTB integrity assay of the dsCREB group. Biotin (red signal) flows into the lumen and indicates damage to HTB (**d**–**f**), while the HTB was intact in dsGFP group (**a**–**c**). (**G**) The distribution of cell junction proteins after the knockdown of es-CREB. (**a**,**e**) α-catenin with green, (**b**,**f**) β-catenin with red, (**c**,**g**) ZO-1 with red and (**d**,**h**) Pinin with red signals. The white arrow pointed the differences of distribution of junctional proteins between the dsGFP and dsCREB groups. The white dotted lines in (**Cd**–**Cf**,**Fb**,**Fe**) showed the boundaries of seminiferous lumens, and the white arrows in (**Ch**,**Ff**) pointed to the permeated biotin (red signal) in seminiferous lumens. * *p* < 0.05 indicates a significant difference between two groups; ** *p* < 0.01 indicates an extremely significant difference; *** *p* < 0.001 indicate an extremely significant difference. ‘ns’ indicates that there was no significant change. The bars in (**C**,**D**,**F**,**G**) represent 50 μm.

To evaluate whether the two MMPs function in the testis of *E. sinensis* and are associated with the expression of protein junctions, we knocked down es-MMP14 and es-MMP21 in the crabs, respectively. Similar changes in protein expression were observed between the dsMMP14 and dsMMP21 groups, and the expression of β-catenin, ZO-1 and Pinin increased significantly; however, α-catenin clearly decreased (Figure 6A,B). In addition, the distribution of junction proteins did not alter after MMP knockdown (Figure 6D). The results indicate that β-catenin, ZO-1 and Pinin may be target proteins degraded by es-MMP14 and es-MMP21 and that the expression of α-catenin was not related to es-MMPs.

**Figure 7:**
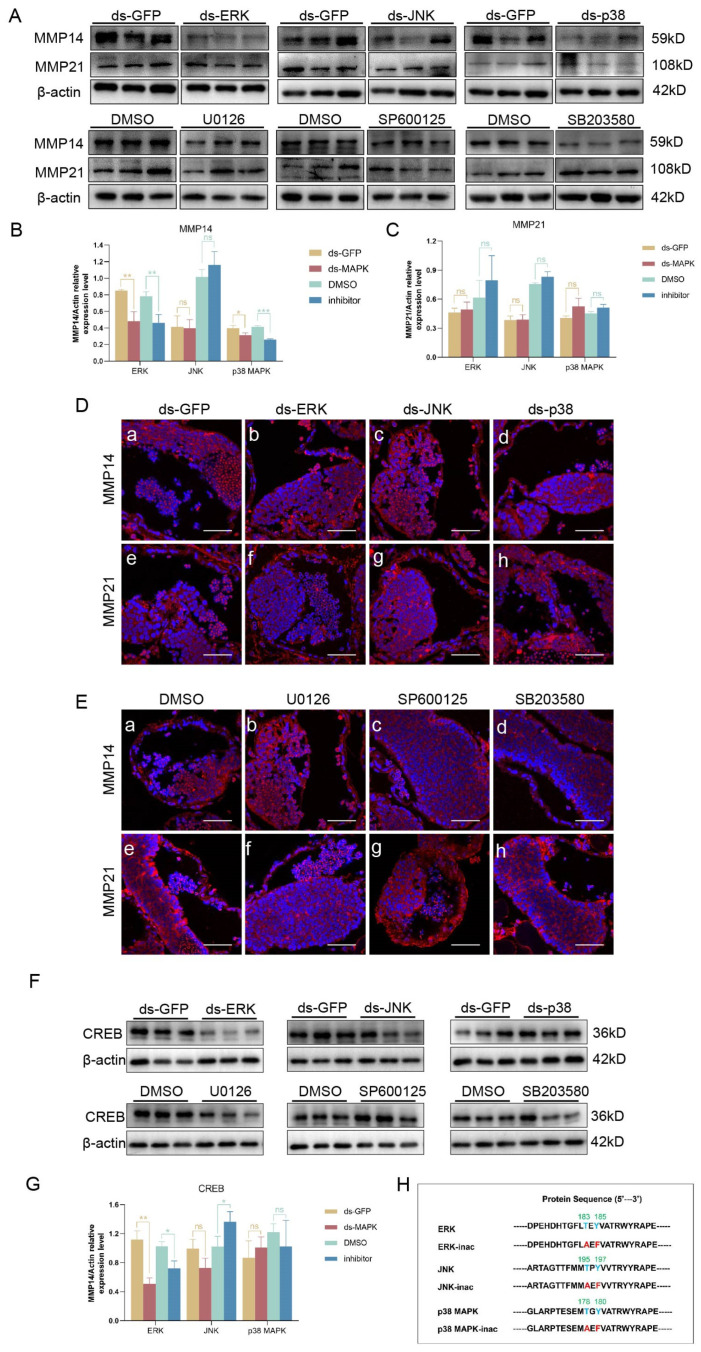
Changes in es-MMP14, es-MMP21 and es-CREB after knockdown or inhibition of the MAPK signaling pathway. (**A**) Expression levels of es-MMP14 and es-MMP21 in the dsMAPK and inhibit-MAPK groups. (**B**) The analyses of es-MMP14 by using ImageJ2. (**C**) The analyses of es-MMP21 by ImageJ2. After the knockdown or inhibition of ERK and p38 MAPK, es-MMP14 reduced but es-MMP21 did not change, and the deficiency or inhibition of JNK did not change either es-MMPs. (**D**) Changes in the distribution of es-MMP14 and es-MMP21 in the dsMAPK groups. (**a**,**e**) were the dsGFP group, (**b**,**f**) were the dsERK group, (**c**,**g**) were the dsJNK group and (**d**,**h**) were the dsp38 MAPK group. (**E**) Change in the distribution of es-MMP14 and es-MMP21 in the MAPK inhibitor treatment groups. (**a**,**e**) were the DMSO group, (**b**,**f**) were the U0126 group, (**c**,**g**) were the SP600125 group and (**d**,**h**) were the SB203580 group. the red signals represent es-MMP14 or es-MMP21. These parameters were not different from those of the control groups. (**F**) Expression level of es-CREB in the dsMAPK or inhibit-MAPK groups. (**G**) The results from (**F**) were examined by ImageJ2. The level of es-CREB decreased only when ERK was deleted or inhibited. (**H**) Detailed information on the site-specific mutagenesis of ERK, JNK and p38 MAPK. * *p* < 0.05 indicates a significant difference between two groups; ** *p* < 0.01 indicates an extremely significant difference; *** *p* < 0.001 indicate an extremely significant difference. ‘ns’ indicates that there was no significant change. The bars in (**D**,**E**) represent 50 μm.

Next, we further explored whether es-MMP14 and es-MMP21 function in the MAPK signaling pathway. We tested the level of es-MMPs. According to the Western blotting results, the deletion or inhibition of ERK or p38 MAPK decreased the expression of es-MMP14 compared with that in the control groups, but there was no change in es-MMP21; moreover, neither deletion nor inhibition of JNK changed the two kinds of es-MMPs (Figure 7A–C). Because the distribution of MMPs is important for their degradation functions, we detected their localization in the dsMAPK and inhibit-MAPK groups by immunofluorescence, and we did not find a significant difference between the control and treatment groups (Figure 7D,E). These findings suggest that ERK and p38 MAPK influence the expression of es-MMP14 rather than its functional position. In conclusion, es-MMP14 rather than es-MMP21 might function downstream of ERK and p38 MAPK and degrade ZO-1 and Pinin.

### 2.6. ERK Changes the Expression Level of ZO-1 through es-CREB in the Testis of E. sinensis

There are two processes involved in regulating the expression of intercellular junctions—the transcription and translation of genes and the degradation of proteins. Based on the above results, we confirmed that ERK and p38 MAPK mediate the degradation of junction proteins through es-MMP14. Next, we further explored how the MAPK signaling pathway influences cell junction expression via gene transcription. Es-CREB is a widely studied transcription factor that is also regulated by the MAPK signaling pathway in mammals.

**Figure 8:**
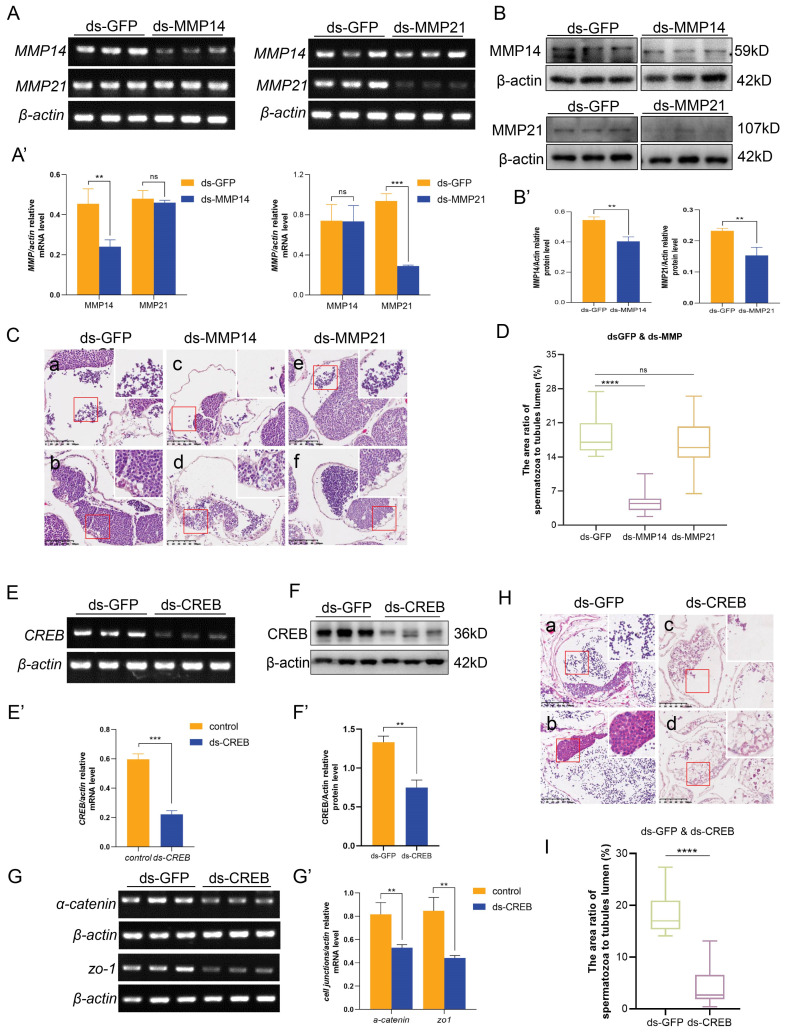
Efficiency of knockdown and morphology of the testis after injection of dsRNA against es-MMP14, es-MMP21 and des-CREB in vivo, respectively. (**A**) sqPCR results showing the efficiency of *es-MMP14* and *es-MMP21* knockdown. (**A′**) We used ImageJ2 to analyze the data in (**A**). (**B**) Protein levels of es-MMP14 and es-MMP21 after the deletion of es-MMP14 and es-MMP21, respectively. (**B′**) Analysis of the es-MMP14 and es-MMP21 protein levels. Both the dsMMP14 and dsMMP21 groups exhibited significant and unique knockdown. (**C**) Morphology of the testis shown by H&E staining. (**a**,**e**) had substantial mature sperm, while (**c**) in the dsMMP14 group, there were fewer spermatozoa in the evacuation zone. The germinal zones of (**b**,**f**) were regular from the control and dsMMP21 groups but disordered in (**d**) from the dsMMP14 group. The red squares were the representative views and we enlarged them in the top of right. (**E**) mRNA level of *es-CREB* after the deletion of es-CREB compared with that in the control group. These results were examined by ImageJ2 and are shown in (**E′**). (**F**) Protein level of es-CREB after its deletion, and (**F′**) shows the analyzed results. (**G**) sqPCR was used to measure the mRNA levels of *α-catenin* and *ZO-1* after knocking down es-CREB, and the analyzed results in (**G′**) revealed that the transcription of *α-catenin* and *ZO-1* was reduced significantly compared with control. (**H**) H&E staining results in the dsCREB group. Vacuolization was observed in the germinal zone (**b**,**d**), and fewer spermatozoa were observed in the evacuation zone (**a**,**c**). The red squares were the representative views and we enlarged them in the top of right. (**D**,**I**) The area ratio of mature sperm to the evacuation zone of (**C**) and (**H**), respectively. ** *p* < 0.01 indicates an extremely significant difference; *** *p* < 0.001 and **** *p* < 0.0001 indicate an extremely significant difference. ‘ns’ indicates that there was no significant change. The scale bars are 100 μm in (**C**,**H**).

To determine the relationship between es-CREB and MAPK, we interfered with es-CREB in *E. sinensis* testes and detected cell junctions. The expression of α-catenin and ZO-1 decreased significantly (Figure 6E), while the distribution of α-catenin and Pinin became dotted, and ZO-1 was irregular (Figure 6G). We hypothesized that es-CREB is a transcription factor of α-catenin and ZO-1 and that es-CREB deficiency decreases their transcription. As expected, sqPCR showed that the mRNA levels of α-catenin and ZO-1 indeed decreased after knocking down es-CREB (Figure 8G). Therefore, does the MAPK signaling pathway influence the transcription levels of α-catenin and ZO-1 through es-CREB? After knocking down or inhibiting the MAPK pathway, we detected that es-CREB was significantly reduced only in the dsERK and U0126 treatment groups (Figure 7F,G). Accordingly, es-CREB functions under ERK and regulates the transcription of ZO-1 but does not function under p38 MAPK to influence the transcription of α-catenin in the *E. sinensis* testis.

### 2.7. ERK and p38 MAPK Influence the Expression and Distribution of Intercellular Proteins When They Are Overexpressed In Vitro

To explore the mechanism by which the MAPK signaling pathway regulates intercellular proteins, we elucidated the influence and relationship between the MAPK pathway and the MMP, CREB and cell junction proteins in vitro. As there are currently no mature crab cell lines available for in vitro experiments, we used the HEK293T cell line, which can be easily transfected for in vitro studies. The activity of the MAPK signaling pathway is more important than its expression level for downstream regulation, and we obtained the nonphosphomimetic MAPK through site-specific mutagenesis, which simulated the inactivity of MAPK (Figure 7H). After transfecting the sequence and nonphosphomimetic sequence of ERK, JNK or p38 MAPK into HEK293T cells, we detected changes in the MMP, CREB and junctional proteins.

**Figure 9:**
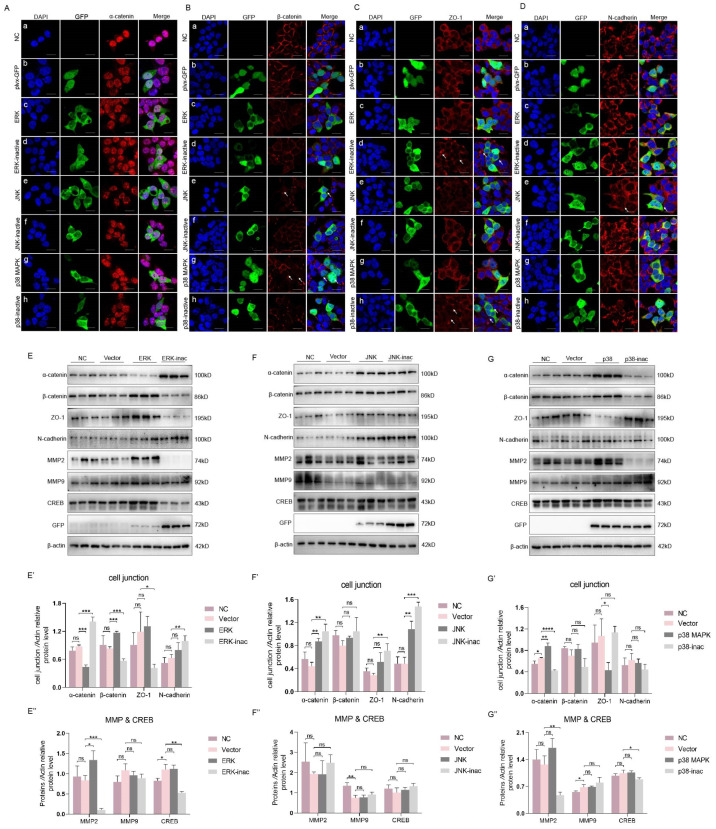
The influence of overexpressed ERK, JNK or p38 MAPK on junctional proteins, MMPs and CREB in vitro. (**A**–**D**) The distribution of α-catenin, β-catenin, ZO-1 and N-cadherin in the negative control, plasmid control, MAPK-overexpressing and MAPK-inac-overexpressing groups of HEK293T cells. (**Aa**–**Ah**) The distribution of α-catenin in NC, GFP, ERK overexpression, ERK-inactive overexpression, JNK overexpression, JNK-inactive overexpression, p38 overexpression, p38-inactive overexpression groups. (**Ba**–**Bh**) The distribution of β-catenin in NC, GFP, ERK overexpression, ERK-inactive overexpression, JNK overexpression, JNK-inactive overexpression, p38 overexpression, p38-inactive overexpression groups. (**Ca**–**Ch**) The distribution of ZO-1 in NC, GFP, ERK overexpression, ERK-inactive overexpression, JNK overexpression, JNK-inactive overexpression, p38 overexpression, p38-inactive overexpression groups. (**Da**–**Dh**) The distribution of N-cadherin in NC, GFP, ERK overexpression, ERK-inactive overexpression, JNK overexpression, JNK-inactive overexpression, p38 overexpression, p38-inactive overexpression groups. The blue signals are DAPI (nucleus), the green signals are GFP, indicating successful overexpression, and the red signals are junctional proteins. The white arrows pointed the changes of distribution of junctional proteins between control and treatment groups. (**E**–**E″**) The protein levels of junctional proteins, MMPs and CREB in ERK-overexpressing and ERK-inac-overexpressing groups in vitro. (**E′**,**E″**) show the analyzed results of (**E**). (**F**–**F″**) and (**G**–**G″**) are similar to (**E**–**E″**). * *p* < 0.05 indicates a significant difference between two groups; ** *p* < 0.01 indicates an extremely significant difference; *** *p* < 0.001 and **** *p* < 0.0001 indicate an extremely significant difference. ‘ns’ indicates that there was no significant change. The bars in (**A**–**D**) represent 20 μm.

Consistent with the findings in vivo, these findings showed that nonphosphomimetic ERK inhibited the expression of ZO-1 by decreasing CREB (Figure 9E), but p38 MAPK through mediating the MMP degraded ZO-1 to reduce its content compared to the nonphosphomimetic p38 MAPK (Figure 9G). In addition, changes in the distribution of cell junctions were detected, and the overexpression of JNK and p38 MAPK resulted in the intermittent localization of β-catenin (Figure 9(Be,Bg)). ZO-1 became uneven in the intercellular spaces when ERK was inactive (Figure 9(Cd)); in addition, the signal of ZO-1 was weaker in the nonphosphomimetic p38 MAPK overexpression group (Figure 9(Ch)). The distribution results were slightly different from our in vivo findings, possibly because of the complicated mechanism involved in *E. sinensis* and the lack of homology between the in vivo and in vitro results.

### 2.8. Loss of es-MMP14 or es-CREB Disrupts HTB Integrity and Spermatogenesis in the Testis of E. sinensis

Although the loss of es-MMP14, es-MMP21 or es-CREB changed junction protein expression, we further illustrate their effects on the integrity of the HTB and spermatogenesis in *E. sinensis*. Western blotting and sqPCR results suggested that es-MMP14, es-MMP21 and es-CREB were expressed at low levels in the knockdown groups, and the efficiencies of knockdown were exclusive and dramatic (Figure 8A,B,E,F). Furthermore, we detected unsuccessful spermatogenesis in the testis and abnormal morphology of the seminiferous tubules in the dsMMP14 and dsCREB groups (Figure 8C,H), which was consistent with our previous description. A large number of spermatozoa were lost in the lumen in the dsMMP14 (4.5%) and dsCREB (4.3%) groups compared with that in the dsGFP group (Figure 8D,I), and their germinal zone also displayed many vacuolizations (Figure 8(Cd,Hd)). However, H&E staining of dsMMP21 revealed regular spermatocytes and spermatids in the germinal zone, and mature spermatozoa were enriched in the tubules (Figure 8(Ce,Cf)). Afterward, we carried out an HTB integrity assay to explore whether the barrier structure still existed. Biotin permeated into the lumen when es-MMP14 or es-CREB was knocked down (Figure 6(Cb,Ce,Ch,F)), while it stopped out of the basement of the tubules when es-MMP21 was deleted (Figure 6(Cc,Cf,Ci)). In summary, es-MMP14 or es-CREB deficiency damages the integrity of HTB in *E.sinensis* and influences the production of spermatozoa.

## 3. Discussion

Although there are some differences in the seminiferous tubule and spermatogenesis process between crustaceans and mammals, a certain structure for protecting spermatogenesis is necessary for them during evolution. The evolutionary mechanisms have not yet been investigated, but more and more research proved the existence of a male reproduction barrier in Echinodermata [45], mollusks [46], nematodes [47], crustaceans [48] and insects [49]. Therefore, we believe that the BTB might be an evolute form of the HTB in invertebrate species, so the functional evolution of conserved genes during species evolution is worth exploring.

### 3.1. MAPK Is Closely Associated with the Reproduction of Male E. sinensis

The MAPK signaling pathway broadly regulates cellular activities, including gene expression, mitosis, metabolism, cell death and migration [50]. Previous studies have shown that ERK1/2 exists at all stages in germ cells and Sertoli cells in mice, and its activity peaks in spermatogonium and preleptotene spermatocytes [51]. ERK plays a crucial role in the regulation of ES dynamics and BTB integrity via TGF-β3, TNF-α and testosterone (T) [52,53]. JNK is an important factor during the development of the testis, especially in the production of healthy spermatozoa from the aspect of apoptosis [54]. In addition, p38 MAPK signaling functions in various processes to modulate male fertility [20], such as the self-renewal and differentiation of spermatogonial stem cells [55], BTB dynamics and the formation of lactic acid [56,57]. Furthermore, the MAPK signaling pathway mediates the disruption of spermatogenesis by PM2.5, accompanied by loose seminiferous tubules and few sperm [58]. Recently, the MAPK signaling pathway has been identified in crustaceans, most of which are involved in immune or antioxidant functions [38,59,60]; others have described its effects on spermatogenesis and the acrosome reaction [39,40]. In our previous research on the mechanism of es-Myosin VI in spermatogenesis in *E. sinensis*, an abnormal MAPK signaling pathway was observed [42], suggesting that the MAPK signaling pathway participates in and plays a key role in this process. Therefore, we investigated the detailed function of the MAPK signaling pathway in spermatogenesis in *E. sinensis*.

Our findings indicated that the expression and activity levels of three key factors in the MAPK signaling pathway were indispensable for spermatogenesis in *E. sinensis*. ERK and p38 MAPK seemed to play more critical roles, as their deletion or inhibition caused more serious defects in seminiferous tubule morphology, and the early germ cells were disorganized in the germinal zone and could not produce mature sperm. These defects are similar to those in mammals when MAPK signaling is abnormal [58]. Although the deletion or inhibition of JNK resulted in vacuolization in the germinal zone, it had no effect on the production of spermatozoa.

### 3.2. Abnormal MAPK Signaling Pathway Impairs the Integrity of HTB through Impacting Intercellular Junctions

Diverse external stimuli and cytokines cause cascade reactions in the MAPK signaling pathway, which participates in various physiological activities by modulating the expression levels of target genes. FSH and T maintain the integrity of the BTB through ERK and its downstream transcription factors (such as CREB and ATF-1), which are related to the expression of junctional proteins in the BTB [11,12]. JNK encodes the transcription of MMPs under TNF-α stimulation and subsequently disturbs TJs [33]. A recent study showed that p38 MAPK interacts with the Wnt/β-catenin and MLCK signaling pathways, regulating TJ expression and BTB integrity in mice, as demonstrated in the TM4 cell line in vitro [36]. We confirmed the existence of HTB in *E. sinensis*, which has a position and function similar to those of the BTB [41]. In addition, the deletion of es-Myosin VI disrupted the integrity of the HTB via changes in the MAPK signaling pathway [42]. Accordingly, we speculated that the MAPK signaling pathway might be associated with the maintenance of HTB integrity.

By performing an HTB integrity assay, we found that a decrease in the content or activity of ERK, JNK and p38 MAPK disturbed the integrity of the HTB. α-catenin, β-catenin, ZO-1 and Pinin are important constituents of HTB, and we detected changes in these proteins to further explore the damage caused by the HTB. However, abnormal expression of these junctions was observed in the ERK- or p38 MAPK-treated groups. Next, the distribution of these junctions between intercellular spaces was also examined, and we found that JNK impacted the HTB by influencing the localization of junctions, which was consistent with our hypothesis, especially for β-catenin and ZO-1. Although the detailed mechanisms are different, the MAPK signaling pathway maintains the integrity of the HTB through the expression and localization of junctional proteins.

### 3.3. ERK and p38 MAPK Affect the Expression Level of Junctional Proteins in HBT Relying on the Degradation of es-MMP14 in the E. sinensis Testis

As the MAPK signaling pathway functions by influencing the expression of target genes, we further explored downstream molecules in the cascade. MMP, disintegrin and metalloproteases (ADAM) reportedly degrade junction proteins in the extracellular matrix (ECM), and the basement membrane outside of the seminiferous tubule is a modified ECM [61]. MMPs are involved in the dynamics of junctions in the testis. The deletion of MMPs causes the disordered formation of the BTB, but superabundant MMPs impair the integrity of the BTB [61,62]. Phosphorylated rpS6 increases the expression of MMP9 in mouse testes and subsequently damages the BTB via the degradation of MMP9 on TJs between Sertoli cells [14]. The expression of MMP2 was downregulated upon exposure to dibutyl phthalate (DBP) because of reduced NF-κB, after which the premature formation of TJs occurred, which impaired spermatogenesis [63]. In addition, MMP2, MMP8 and MMP9 cooperate under microcystin-LR (MC-LR) treatment in response to degrading TJs, and the upregulation of MMP8 is caused by the ERK and JNK signaling pathways and NF-κB [64]. MMP9 is highly expressed after treatment with hexafluoropropylene oxide (HFPO) because of p38 MAPK, after which it degrades occludin in the testis [35]. Considering the regulation of MMPs by the MAPK signaling pathway, we hypothesized that MMPs participate in the maintenance of the HTB via the MAPK signaling pathway in *E. sinensis*.

We detected two types of MMPs in *E. sinensis* testes—es-MMP14 and es-MMP21. Es-MMP14 has been shown to be related to the immune response to external stimuli and disease in *E. sinensis* [44], which is similar to the traditional activation pattern of the MAPK signaling pathway. In mammals, MMP14 is a membrane-type MMP and is also referred to as MT1-MMP [65]. Most studies on MMP14 have focused on cell migration, invasion and degradation of the ECM, but MMP14 is closely related to the activity of MMP2 and MMP9. Pro-MMP2 is secreted by cells without activity; then, it is activated by MMP14 and becomes active MMP2, which programs its degradation function [65,66]. MMP9 can also be activated by MMP14 during the invasion of glioma cells [67]. In addition, TGF-β1 activates ERK1/2 and p38 MAPK as well as the downstream regulation of MMP14 on MMP2 [68]. Furthermore, research in pulmonary artery smooth muscle cells showed that p38 MAPK increased the expression of MMP14 by activating the activity of the transcription factor NF-κB, subsequently regulating MMP2 function. Under electromagnetic radiation, an impaired BTB and low male fertility are caused by reduced binding between MMP14 and MMP2 [69]. These studies provide evidence for the crucial role of MMP14 in maintaining BTB integrity and male fertility. We first identified another es-MMP, es-MMP21, in the present study, but there are few related studies. We found that the deletion of es-MMP14 and es-MMP21 changed the expression pattern of junctional proteins, which seemed to agree with the degradation function of MMPs; in particular, β-catenin, ZO-1 and Pinin were regarded as the degraded target proteins because their expression increased significantly after the deficiency of es-MMPs. Although these junctions are cytoplasmic proteins, MMPs have been shown to be involved in a variety of signaling and homeostatic systems [70]. Although there was no reason for the reduction in α-catenin after MMP knockdown, more complex mechanisms may participate in this process rather than in degradation. Surprisingly, es-MMP14 deletion disrupted the integrity of the HTB, but es-MMP21 did not. We believe that es-MMP21 may function primarily during restructuring of the HTB and that it barely plays a role in the maintenance of the HTB. Next, we detected es-MMPs in the MAPK- deletion or MAPK- inhibition groups to explore the regulation of es-MMPs by the MAPK signaling pathway. ERK and p38 MAPK impacted the expression rather than the distribution of es-MMP14.

To verify the regulation of es-MMPs by the MAPK signaling pathway and the influence of intercellular junctions, we overexpressed ERK, JNK and p38 MAPK or their inactive forms in vitro to simulate normal or nonphosphorylated conditions. MMP2 and MMP9 were examined due to their ability to degrade junctional proteins, and N-cadherin was commonly detected in the ECM. The in vitro results further illustrated that es-MMPs might affect the expression of intercellular junctions downstream of the MAPK signaling pathway, especially ZO-1.

### 3.4. Es-CREB Has an Impact on the Transcription Level of ZO-1 Downstream of ERK in the E. sinensis Testis

The expression level is regulated by not only posttranslational degradation but also modulation of transcription. CREB is expressed in Sertoli cells and is essential for spermatogenesis in rats, and inactive CREB in Sertoli cells causes apoptosis of the spermatogonium and reduces the number of mature sperm [71]. CREB is activated by ERK and cAMP-PKA in Sertoli cells under the regulation of T and FSH, respectively, and subsequently adjusts gene transcription [72]. In the present study, es-CREB was cloned from *E. sinensis* testes, and we found that it influenced the mRNA levels of *α-catenin* and *ZO-1*. The deletion of es-CREB strongly disrupted the integrity of the HTB, which was similar to the results showing an abnormal MAPK signaling pathway. Furthermore, we detected reduced es-CREB in the ERK deletion and inhibition groups but did not observe changes in the levels of JNK and p38 MAPK. These findings suggested that ERK impaired the HTB through the modulation of ZO-1 transcription by es-CREB. Similarly, we obtained similar in vitro experimental data, and the activity of ERK and CREB and the expression of ZO-1 were positively correlated.

### 3.5. The Cytoskeleton Might Be Involved in the MAPK Signaling Pathway for HTB Integrity Maintenance

However, whether and how the MAPK signaling pathway affects the integrity and function of the HTB in *E. sinensis* remain largely unknown. For example, es-MMP14 and es-CREB are downstream of ERK but oppositely regulate ZO-1, and transcription and translation are preconditions for proteins. When transcription is hindered, decreased degradation cannot rescue a low level of proteins. In addition, whether the deficiency of es-MMPs reduces the expression of α-catenin also needs to be explored. In addition to ERK and p38 MAPK, we seemed to ignore the crucial role of JNK in maintaining the integrity of the HTB. JNK controls the epithelial barrier by phosphorylating cofilin and then destroying F-actin at tricellular contacts [73]. Moreover, JNK is associated with the RhoA/ROCK signaling pathway, which phosphorylates MLC [74,75], and phosphorylated MLC represents an unstable microfilament. However, the normal distributions of α-catenin, β-catenin and ZO-1 anchor to the cytoskeleton, especially F-actin. The instability of F-actin cannot provide effective anchor sites for junctional proteins, resulting in a destroyed BTB. In the present study, we found that knockdown of the inhibition of JNK changed the distributions of intercellular proteins; therefore, it is necessary for us to investigate whether JNK maintains the integrity of the HTB through the cytoskeleton in *E. sinensis* in the future.

## 4. Materials and Methods

### 4.1. Experimental Animals

We bought crabs from the Chongming Research Base of Shanghai Ocean University for our experiments; the average weight of the crabs was 100 ± 10 g. The crabs were cultured at 22–28 °C and given sufficient oxygen via oxygen pumps. We changed the water and fed them with commercial feed (Ankang Crab, Alpha Feed, Shenzhen, China) every day. Fifteen crabs per group were acclimated for one week before the interference and inhibition experiments.

We purchased three-week-old female ICR mice (SPF) from Shanghai SLAC Laboratory Animal Co., Ltd. (Shanghai, China), for performing the polyclonal antibodies preparation experiments. Before the experiment, we fed the mice with commercial feed (Lab Rat and Mouse Diet, Xietong Pharmaceutical Bio-engineering Co., Ltd., Nanjing, China) for one week at the Laboratory Animal Center of Zhejiang University.

All the experimental animals were approved by the Animal Experimental Ethical Inspection of the First Affiliated Hospital, Zhejiang University School of Medicine, with approval number “2021–557”.

### 4.2. Cell Culture

The human embryonic kidney (HEK) 293T cell line was purchased from Byotime (Shanghai, China, C6008) and used in our study. HEK 293T cells were cultured in Dulbecco’s modified Eagle’s medium (DMEM) (MeilunBio, Dalian, China), supplemented with 10% fetal bovine serum (FBS) (Tianhang, Hangzhou, Zhejiang, China) and 1% penicillin/streptomycin (MeilunBio), and the cells were incubated under 5% CO_2_ at 37 °C.

### 4.3. Cloning of es-MMP21 and es-CREB in E. sinensis

We obtained the total RNA from crab testes using RNAiso (Takara, Dalian, China). Then, we used PrimeScipt^TM^ RT Master Mix (Takara) to reverse transcribe total RNA and obtained cDNA from crab testes for further experiments. According to the transcriptome library of *E. sinensis*, we designed primers using Primer 5.0 and obtained cDNA fragments of *es-MMP21* and *es-CREB* (Table 1). The system and program PCR are the same our previous description [42]. The pMD19-T-vector (Takara) and *Escherichia coli* DH5α (Takara) were used for cloning and amplification. Finally, the clone bacteria solution was sequenced by Tsingke (Beijing, China). We spliced the fragments and obtained the gene coding sequence (CDS).

### 4.4. Bioinformatics Analysis of es-CREB, es-MMP14 and es-MMP21

We used an online tool (https://web.expasy.org/translate/, accessed on 1 August 2023) to translate the CDSs of *es-MMP21* and *es-CREB*, and another online tool, SMART (http://smart.embl-heidelberg.de/, accessed on 11 August 2023), was used to predict their secondary structures. We tested the conservation of *es-MMP14* and *es-MMP21* with other species using the National Center for Biotechnology Information (NCBI) Protein BLAST website (https://blast.ncbi.nlm.nih.gov/Blast.cgi, accessed on 15 August 2023), and we used the online tool SMS v2 for multiple sequence alignments. Next, we built a phylogenetic tree of *es-CREB*, *es-MMP14* and *es-MMP21* by using the software MEGA 5.0. The following protein sequences from other species were downloaded from NCBI: *Danio rerio* MMP14b precursor (NP_919395.1), *D. rerio* MMP21 (ALF36875.1), *Homo sapiens* MMP14 preproprotein (NP_004986.1), *H. sapiens* MMP21 (AAM78033.1), *H. sapiens* CREB (AAQ24858.1), *Hyalella azteca* MMP14 (XP_018010335.1), *Mus musculus* MMP14 (AAB86602.1), *M. musculus* MMP21 (AAI11523.1), *M. musculus* CREB1 (NP_034082.1), *Penaeus chinensis* MMP14-like (XP_047479092.1), *P. chinensis* CREB1-like (XP_047495257.1), *Penaeus monodon* MMP14-like (XP_037802224.1), *P. monodon* MMP21-like (XP_037777210.1), *P. monodon* CREB1-like (XP_037789700.1), *Penaeus vannameiv* MMP14-like (XP_027228337.1), *P. vannamei* MMP21-like (XP_027235723.1), *P. vannamei* CREB3 (ATY51984.1), *Portunus trituberculatus* MMP14 (MPC29569.1), *P. trituberculatus* MMP21 (MPC09945.1), *Xenopus tropicalis* MMP14 precursor (NP_001025559.1), *X. tropicalis* MMP21 (XP_002932698.2), *X. tropicalis* CREB1-like (AAH67956.1), *Caerostris darwini* MMP21 (GIY90400.1), *Homarus americanus* MMP21-like (KAG7158678.1), *Nephila pilipes* MMP21 (GFU45318.1), *Procambarus clarkia* CREB1-like (AGZ84433.1), *Procambarus clarkia* CREB (XP_045616617.1), *Homarus americanus* CREB1-like (XP_042208840.1), *Penaeus japonicus* CREB1-like (XP_042882462.1).

### 4.5. Semiquantitative Real-Time PCR (sqPCR)

The efficiency of knockdown was determined by sqPCR, and the primers used are listed in Table 1. The composition and program of the PCR system were the same as those for the gene clone, but the optimal number of cycles for each gene was determined through an experiment with a gradient of cycle numbers. β-actin served as the internal control in this study. The experimental and control groups with different treatments were relatively independent. There were at least three individuals selected for control or experimental groups, and each result was repeated three times.

### 4.6. Antibodies

The es-ERK antibody was purchased from Beyotime (Shanghai, China, AF0144). We prepared antibodies against es-JNK, es-p38 MAPK, es-MMP14, es-MMP21 and es-CREB for the present research (Supplementary Figure S4). To obtain the fragments that encoded the peptides of the above genes, the composition and PCR system used were the same as those used for the gene cloning; primers with homologous arms of pET-28a (Takara, Dalian, China) and restriction enzyme cutting sites of BamH I and EcoR I are listed in Table 1. We used a ClonExpress II One Step Cloning Kit (Vazyme, Nanjing, China) to clone the fragments into pET-28a, which can express the polypeptide. Next, Rosetta-competent cells were used to express the fragments. The Rosetta strain was cultured in LB medium supplemented with 30 μg/mL kanamycin at 37 °C at a speed of 200 rpm, and 0.5 mM isopropyl-β-d-thiogalactoside (ITPG) was added to the medium when the OD600 of the strain reached 0.4–0.6. Then, the strain was cultured overnight at 37 °C and at 200 rpm. On the second day, we used PBS with phenylmethyl sulfonyl fluoride (PMSF) to extract the Rosetta strain and obtained the peptide fragments. Then, a His-tagged Protein Purification Kit (Inclusion Body Prodtein) (CoWin Biosciences, Taizhou, Jiangsu, China) was used to purify the target peptides. Before we injected the mice with the purified proteins, they were mixed with Freund’s adjuvant (Sigma–Aldrich, St. Louis, MO, USA) equally. The first injection consisted of complete adjuvant, while the other times, instead of incomplete adjuvant, injection continued for one month and once a week. One week after the last injection, we gained mouse blood serum as a polyclonal antibody.

### 4.7. Plasmid Construction

We constructed the CDSs of es-ERK, es-JNK and es-p38 MAPK with pLVX-GFP victor, and we constructed the corresponding nonphosphorylation mutant sequences with pLVX-GFP using overlap PCR. Threonine (ACT in 183) and tyrosine (TAT in 185) were mutated to alanine (GCT) and phenylalanine (TTT) in ERK-inac-pLVX-GFP, respectively; threonine (ACA in 195) and tyrosine (TAT in 197) were mutated to alanine (GCA) and phenylalanine (TTT) in JNK-inac-pLVX-GFP, respectively; threonine (ACT in 178) and tyrosine (TAC in 180) were mutated to alanine (GCT) and phenylalanine (TTC) in p38 MAPK-inac-pLVX-GFP, respectively.

### 4.8. Cell Transfection

HEK 293T cells were transfected with Lipo8000 (Beyotime). We transfected cells in 6-well cell culture plates for Western blotting and in 24-well cell culture plates for cell slipper experiments and IF. One day before transfection, the cells were seeded at 70–80% confluence at transfection, the plasmid was diluted (2.5 μg in 6-well plates and 500 ng in 24-well plates) in OPTI-MEM (125 μL in 6-well plates and 25 μL in 24-well plates), and, then, Lipio8000 (4 μL in 6-well plates and 0.8 μL in 24-well plates) was added. This mixture was mixed gently, and the mixture was added to the cells in fresh medium. After 4–6 h of transfection, the cell medium was exchanged. Further, 24 to 48 h after transfection, proteins were extracted or IF was performed.

### 4.9. Western Blotting

The fresh crab testis samples were homogenized in RIPA lysis buffer (Beyotime), and the cells were lysed in cell lysis buffer for Western and IP (Beyotime), all supplemented with protease and phosphatase inhibitors (Beyotime). The method of protein preparation was the same as our previous description [42]. SDS–PAGE gels were used to separate the same content of proteins and subsequently transferred to PVDF membranes (Merck, Shanghai, China). After blocking the transferred membranes, the membranes were incubated with the primary antibodies overnight at 4 °C (anti-ERK at 1:500, Beyotime; anti-ACTB at 1:5000, Sangon Biotech, Shanghai, China and other antibodies prepared at 1:1000). The dilution rate of the secondary antibody and following steps refer to our previous study [42]. The experimental and control groups with different treatments were relatively independent. There were at least three individuals selected for control or experimental groups, and each result was repeated three times.

### 4.10. Immunofluorescence of MMP and Membrane Costaining

Paraformaldehyde (PFA, 4%) was used to fix the freshly dissected crab testes for 24 h, and then 15% and 30% sucrose was used to dehydrate the tissue. After 24 h, the testes were embedded in O.C.T. and frozen at −20 °C. Using a cryostat microtome (Thermo Scientific, HM525NX, Waltham, MA, USA), we cut the frozen tissue blocks into 8 μm thick sections. The sections were defrosted in PBS for 10 min, and then cellular immune permeable fluid (Saponin, Beyotime) was used to permeabilize the sections for 20 min. Next, 1% BSA/PBS was used to block the sections for 1 h at room temperature. After that, we used primary antibodies (against MMP14 and MMP21, 1:50, 1% BSA/PBS) to incubate the sections overnight at 4 °C. On the second day, we used PBS to wash the sections and incubated the sections with Alexa Fluor 555-labeled goat anti-mouse IgG (1:500 diluted in 1% BSA/PBS) (Beyotime) as the secondary antibody for 1 h at room temperature. The DIO (400×) and staining enhancer were diluted with staining buffer to compose a working fluid for membrane staining (Beyotime). Then, we used this staining fluid to stain the membranes of the sections for 20 min at 37 °C. After the sections were washed with PBS, we stained the nuclei for 10 min by using DAPI (Beyotime) at room temperature. Finally, the sections were sealed with antifade mounting medium, and a laser scanning confocal microscope (FV3000, Olympus, Tokyo, Japan) was used to observe signals.

### 4.11. Immunofluorescence

The freshly dissected crab testes were fixed with 4% PFA, paraffin-embedded tissues, and 5 μm paraffin-embedded sections were obtained from Haoke Biotechology Company (Hangzhou, China). The sections were dewaxed and treated as in our reported method [42]. The primary antibodies were diluted in 1% BSA/PBST (1:50) (the secondary antibodies: Alexa Fluor 555 or 488-labeled goat anti-mouse IgG, 1:500, Beyotime). Then, we used the laser scanning confocal microscope (FV3000, Olympus) to detect the signals after staining with DAPI (Beyotime). The experimental and control groups with different treatments were relatively independent. There were at least three testis sections from individuals selected for control or experimental groups to perform immunofluorescence.

The transfected cells in 24-well plates on small glass lenses were washed with PBS three times; 4% PFA was used to fix the cells for 20 min, and 0.1% PBST (Triton X-100 in PBS) was used for permeabilization. Afterwards, the cells were washed with PBS three times for 5 min each. After blocking with 1% BSA/PBS for 1 h at room temperature, the cells were incubated with primary antibodies at 4 °C overnight. On the second day, we used the Alexa Fluor 555 (diluted with 1% BSA/PBS) to incubate the cells as a secondary antibody for 1 h at room temperature, after which we used a laser scanning confocal microscope (FV3000, Olympus) to examine the signals after staining with DAPI (Beyotime). There were at least three wells of every control or overexpressed group to perform immunofluorescence.

### 4.12. RNA Interference

We contrasted plasmids for expressing dsRNA to silence *es-ERK*, *es-JNK*, *es-p38 MAPK*, *es-MMP14*, *es-MMP21* and *es-CREB*. The primers used for each gene are listed in Table 1, and the sequences of the homologous arms of the L4440 vector and the restriction site of Sma I/Kpn I were used. The segments were subsequently cloned and inserted into L4440 to construct the recombinant plasmids, which were subsequently transformed into HT115 (DE3) competent cells (Weidi Biotechnology, Shanghai, China) that could express dsRNA. The resulting dsRNA was dissolved in diethylpyrocarbonate (DEPC) H_2_O. Every group was injected with 200 μg of dsRNA every time, and the ds-GFP-injected group served as the control. We injected dsRNA every three days for a total of five injections. We obtained the freshly dissected testes of the crabs two days after the last injections.

### 4.13. Hematoxylin–Eosin (H&E) Staining

We obtained the paraffin sections from Haoke Biotechology Company (Hangzhou) via the same methods used for immunofluorescence. The sections were dewaxed and stained as in our reported method [42]. Finally, the mounted sections were scanned by Haoke Biotechology Company (Hangzhou). The experimental and control groups with different treatments were relatively independent. There were at least three testis sections from individuals selected for control or experimental groups to perform H&E staining, and the data were counted from at least thirty seminiferous tubules of different crabs.

### 4.14. Biotin-Streptavidin Immunofluorescence

We injected the crabs for further HTB integrity assays with 1 mg of biotin. After dissection, we obtained paraffin sections from Haoke Biotechology Company (Hangzhou), as described for immunofluorescence. The sections were dewaxed, fixed, stained and observed as in our reported method [42]. The experimental and control groups with different treatments were relatively independent. There were at least three testis sections from individuals selected for control or experimental groups to perform experiments.

### 4.15. Treatment

U0126, SP600125 and SB203580 (Beyotime) were used to inhibit the phosphorylation of ERK, JNK and p38 MAPK in the MAPK signaling pathway, and DMOS was used as the control. We injected the crabs with inhibitors (5 mg/kg), every two days, five times (for a total of ten days). On the third day after the last injection, we dissected the fresh crabs for further experiments.

### 4.16. Statistical Analysis

We analyzed our data by using ImageJ2 and GraphPad Prism 8.0 (GraphPad, Boston, America). Significant differences were analyzed by unpaired two-tailed Student’s *t*-tests. * *p* < 0.05 indicates a significant difference between two groups; ** *p* < 0.01 indicates an extremely significant difference; *** *p* < 0.001 and **** *p* < 0.0001 indicate an extremely significant difference. ‘ns’ indicates that there was no significant change.

## 5. Conclusions

**Figure 10:**
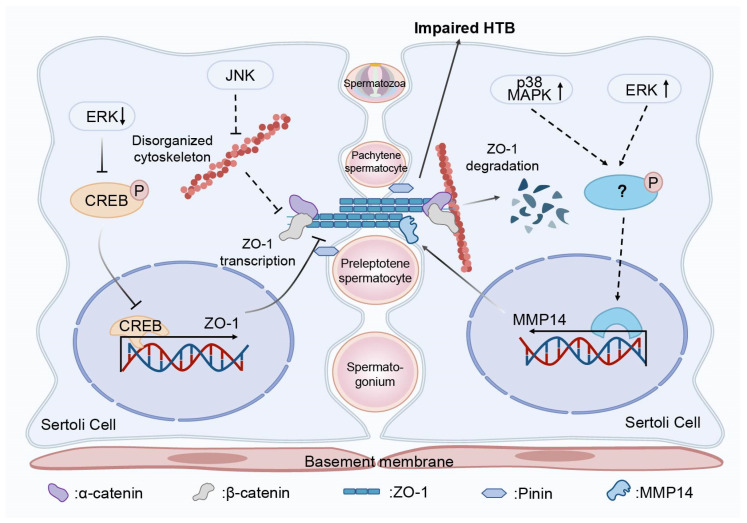
Model of the MAPK signaling pathway functions in regulating the integrity of the HTB and spermatogenesis in *E. sinensis*. ERK and p38 MAPK modulate the expression of es-MMP14, which can degrade junctional proteins, such as ZO-1. In addition, es-CREB may regulate the transcription level of ZO-1 as a transcription factor downstream of ERK. JNK participates in the maintenance of the HTB integrity via the distribution of junctional proteins, which is decided by cytoskeleton. Normal MAPK signaling pathway is critical for the functional HTB and spermatogenesis in *E. sinensis*.

In brief, our findings illustrated the essential role of the MAPK signaling pathway in the HTB of *E. sinensis*. The content and activity of ERK, JNK and p38 MAPK are important, and deficiency or inhibition of any one of them results in an impaired HTB and unsuccessful spermatogenesis, including the formation of vacuolation in the germinal zone and the loss of mature spermatozoa. Furthermore, we found that es-MMP14 was involved in this defect downstream of ERK and p38 MAPK through the degradation of intercellular junctions, such as Pinin and ZO-1, in the testis. In addition, es-CREB regulated the transcription of ZO-1 downstream of ERK. However, JNK did not influence the integrity of the HTB by changing the protein expression but did impact the distribution of junctional proteins in the HTB (Figure 10). We first detected the molecular mechanisms of the MAPK signaling pathway in intercellular junctions in *E. sinensis* testes, which might provide evidence for further research on the roles of the HTB and MAPK signaling pathway in spermatogenesis in crustaceans. However, more factors related to the impact of the MAPK signaling pathway on the HTB other than intercellular junctions should be explored.

## Figures and Tables

**Table 1:** The primer sequences used in the present study.

Primers	Primer Sequence (5′-3′)		Purpose
	Forward	Reverse	
MMP21-1	GAAAGTCCTTCCAAAGCC	TTATCCTCAGTAGGTGCCC	Clone
MMP21-2	GTGAGGTGCCAGGAAACGA	CGTCATACCCGTAGACCTTC	Clone
MMP21-3	ATGGCTACTTGGACTGTGTG	TCCTGATGTGAATCTGGCT	Clone
MMP21-nested-outer	GTTTGAGTGGGGTGGTCTGCCTG		3′ RACE
MMP21-nested-inner1	ACGCACGCAAGACATTCACCTGG		3′ RACE
MMP21-nested-inner2	AATGGACGCATCATCCCCAACAAC		3′ RACE
CREB-1	CATCTTCCAGTAGGAGTTCG	TGCTGCTTCCCTGTTTTT	Clone
CREB-2	TCTCAGCCCTCAAACTCA	CAGTGGATGTGTATAAGGGTT	Clone
ERK-RT	TGAAGCCATCAAACCTCCTC	ATGCAGCCCACAGACCAA	sqPCR
JNK-RT	AAAACCGTCCCCGCTACC	CCTCCGCTTCATCATACCAAA	sqPCR
p38 MAPK-RT	TGTCTCCTGTGGGCTCCG	CCGATGATATTCTCGTGGTCC	sqPCR
MMP14-RT	AGGCACCAACCTCTTCCA	TCGTCTTCGTCCAGACTGAA	sqPCR
MMP21-RT	AGGGCACCTACTGAGGATAA	GCGACGCTTAGAGTCAATGTA	sqPCR
CREB-RT	TATCAGACAAGGGCGACG	GCAGCAACTGGGACAATG	sqPCR
α-catenin-RT	TTGTTGGCGTGCTCAGTG	CATCCTCCAAAATGTGACTCTC	sqPCR
zo-1-RT	TGTCAGAAACTAAGCCCGT	TAGGCACAATAGGTGGTTTC	sqPCR
β-actin-RT	CGAGGCTACACCTTCACGAC	ACGCGGCAGTGGTCATTT	sqPCR
JNK-AB	cagcaaatgggtcgcggatccTATGTTGAAAACCGTCCCCG	ttgtcgacggagctcgaattcGCAGCAGCATTAGTGGCAGTT	Prokaryotic expression
p38 MAPK-AB	cagcaaatgggtcgcggatccATTTTGGACTTTGGGCTGGC	ttgtcgacggagctcgaattcGCAATAACTGTAGGCTTGGGTTG	Prokaryotic expression
MMP14-AB	cagcaaatgggtcgcggatccAACCTCTTCCAAGTAGCCGCC	ttgtcgacggagctcgaattcAAGTAGATTTTCCCGTTTCCGG	Prokaryotic expression
MMP21-AB	cagcaaatgggtcgcggatccCCCACCGTTGGGTTACTGTCG	ttgtcgacggagctcgaattcCCGATGCTGATGTCAACCGTG	Prokaryotic expression
CREB-AB	cagcaaatgggtcgcggatccCAGCCAAGTGTCATCCAGAGC	ttgtcgacggagctcgaattcGCAGCAACTGGGACAATGAAC	Prokaryotic expression
ERK-ds-1	gtgacgcgtggatcccccgggATCCCCATTTGAGCACCAGA	ctatagggcgaattgggtaccTTGGACAACATCTCAGCAAGGA	dsRNA
JNK-ds-1	gtgacgcgtggatcccccgggGCCACGCCCAGCAACTCT	ctatagggcgaattgggtaccAATGAAGATGCTTGATGCCACA	dsRNA
JNK-ds-2	gtgacgcgtggatcccccgggTGTGGCATCAAGCATCTTCATT	ctatagggcgaattgggtaccCCTCCGCTTCATCATACCAAA	dsRNA
p38 MAPK-ds-1	gtgacgcgtggatcccccgggAAGATTTTGGACTTTGGGCTGG	ctatagggcgaattgggtaccTAGGTAGGGATGGGCAAGGG	dsRNA
MMP14-ds-1	gtgacgcgtggatcccccgggAGGCACCAACCTCTTCCAAGT	ctatagggcgaattgggtaccCCACGCCGTCATCTGTAAGC	dsRNA
MMP14-ds-2	gtgacgcgtggatcccccgggACCAAGCCCAATGCGATAAC	ctatagggcgaattgggtaccAGTGAGCGTCTCCTCCGTAGATT	dsRNA
MMP21-ds-1	gtgacgcgtggatcccccgggAGGGCACCTACTGAGGATAAATCA	ctatagggcgaattgggtaccCTCCTGATGTGAATCTGGCTTGG	dsRNA
MMP21-ds-2	gtgacgcgtggatcccccgggGGGGTGGAATGACCGAAAGC	ctatagggcgaattgggtaccCGATGTTGTTGGGGATGATGC	dsRNA
CREB-ds-1	gtgacgcgtggatcccccgggAACAGCCTTCTCAGCCCTCAA	ctatagggcgaattgggtaccGCAGCAACTGGGACAATGAAC	dsRNA
CREB-ds-2	gtgacgcgtggatcccccgggAAAACAGCCTTCTCAGCCCTC	ctatagggcgaattgggtaccAGCCACTCAAGTCCCCAGCA	dsRNA
GFP-ds-1	gtgacgcgtggatcccccgggCGACGTAAACGGCCACAAGTT	ctatagggcgaattgggtaccGATGGGGGTGTTCTGCTGGTAG	dsRNA

## Data Availability

Data are contained within the article.

## Supplementary Materials

The following supporting information can be downloaded at: https://www.mdpi.com/article/10.3390/ijms25137361/s1.
