# Supplementary material for: ERK/CREB and p38 MAPK/MMP14 Signaling Pathway Influences Spermatogenesis through Regulating the Expression of Junctional Proteins in Eriocheir sinensis Testis
Source: Int J Mol Sci. 2024 Jul 4;25(13):7361. doi: 10.3390/ijms25137361 (PMC11242087; doi:10.3390/ijms25137361)
Supplement: Supplementary file 1 [file ijms-25-07361-s001.zip › Supplementary Figures S1-S4.pdf]

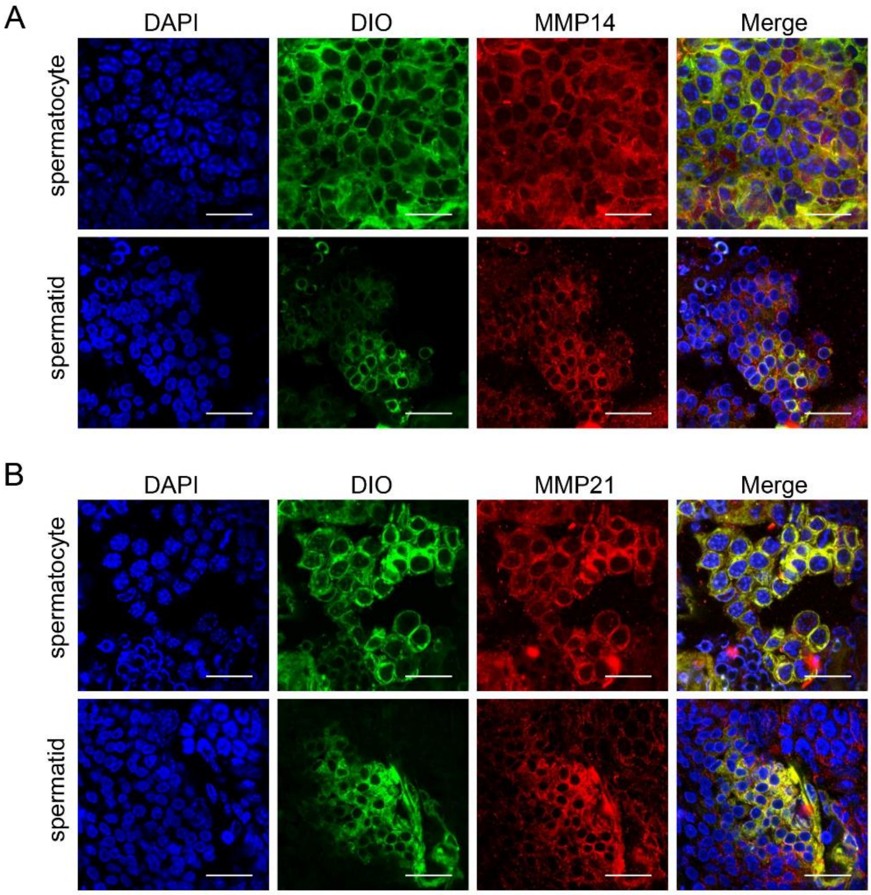

Supplementary Figure S1. The localization of es-MMP14 and es-MMP21 in testis during spermatogenesis in *E. sinensis*. (**A**) In spermatocyte and early spermatids, es-MMP14 (the red signal) distributed in the cytoplasm and cell membrane, it co-localized with membrane tracer (DIO, the green signal). (**B**) Similar to the es-MMP14, es-MMP21 (the red signal) also distributed in the cytoplasm and cell membrane, this could be observed through co-localization with DIO (the green signal). The scale bars represented 20 μm.

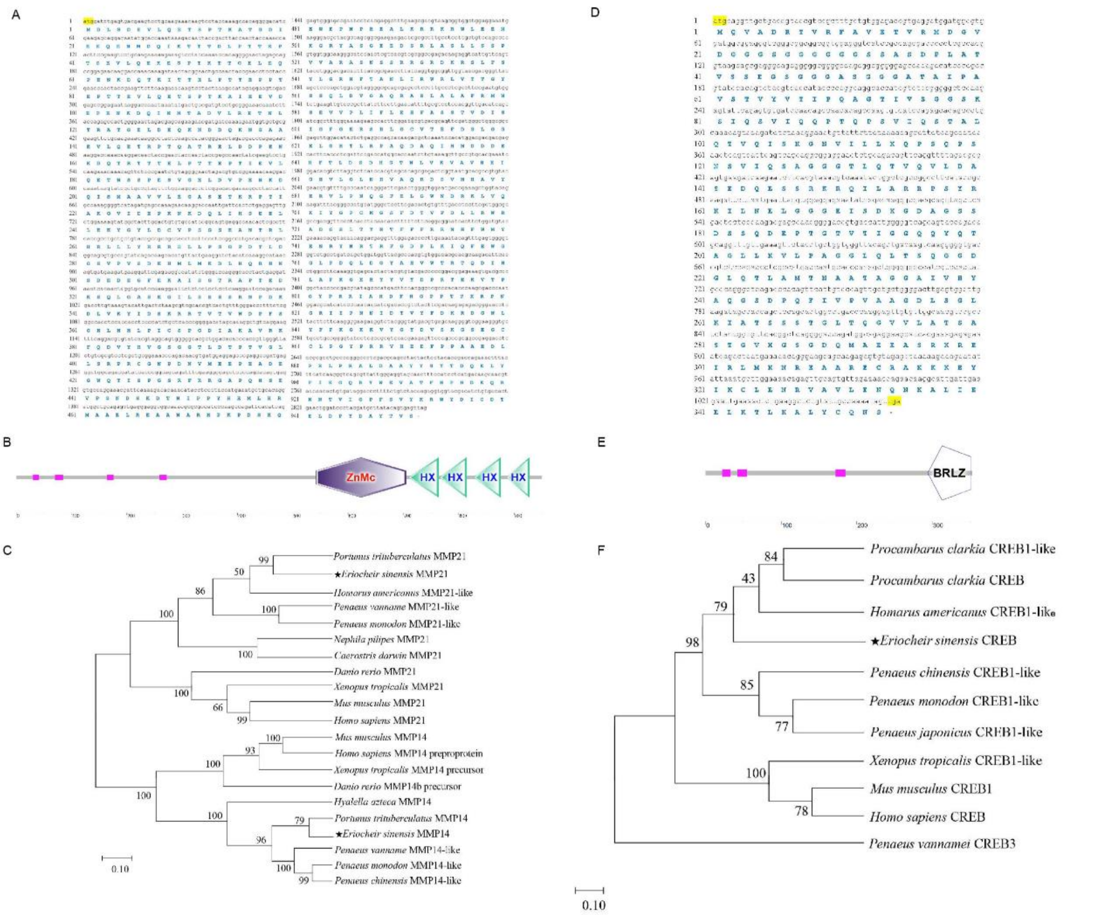

Supplementary Figure S2. The nucleotide and amino sequences, major structural domains and phylogenetic tree of es-MMP21 and es-CREB. (**A**) The CDS of es-MMP21 is 2856 bp which encodes 951 amino acids. The above is nucleotides but the bottom is the amino acids. (**B**) We used NCBI BLAST to predicted the domains of es-MMP21 online, it has a ZnMc classical domain from 542 aa to 705 aa, and four HX domains. The pink rectangles were low complexity regions. (**C**) A phylogenetic tree of MMP14 and MMP21. (**D**) The CDS of es-CREB is 1062 bp which encodes 353 amino acids. (**E**) The primary domain of es-CREB was predicted by NCBI BLAST, it has the BRLZ classical domain (294 – 351 aa). The pink rectangles were low complexity regions. (**F**) The phylogenetic tree of CREB. The follows we list the accession numbers used in the phylogenetic tree: *M. musculus* MMP14 (AAB86602.1), *H. sapiens* MMP14 preproprotein (NP_004986.1), *X. tropicalis* MMP14 precursor (NP_001025559.1), *D. rerio* MMP14b precursor (NP_919395.1), *H. azteca* MMP14 (XP_018010335.1), *P. trituberculatus* MMP14 (MPC29569.1), *P. vannamei*-like (XP_027228337.1), *P. monodon* MMP14-like (XP_037802224.1), *P. chinensis* MMP14-like (XP_047479092.1), *P. trituberculatus* MMP21 (MPC09945.1), *H. americanus* MMP21-like (KAG7158678.1), *P. vannamei* MMP21-like (XP_027235723.1), *P. monodon* MMP21-like (XP_037777210.1), *N. pilipes* MMP21 (GFU45318.1), *C. darwini* MMP21 (GIY90400.1), *D. rerio* MMP21 (ALF36875.1), *X. tropicalis* MMP21 (XP_002932698.2), *M. musculus* MMP21 (AAI11523.1), *H. sapiens* MMP21 (AAM78033.1), *P. clarkia* CREB1-like (AGZ84433.1), *P. clarkia* CREB (XP_045616617.1), *H. americanus* CREB1-like (XP_042208840.1), *P. chinensis* CREB1-like (XP_047495257.1), P. monodon CREB1-like (XP_037789700.1), P. japonicus CREB1-like (XP_042882462.1), X. tropicalis CREB1-like (AAH67956.1), M. musculus CREB1 (NP_034082.1), H. sapiens CREB (AAQ24858.1), P. vannamei CREB3 (ATY51984.1).

A

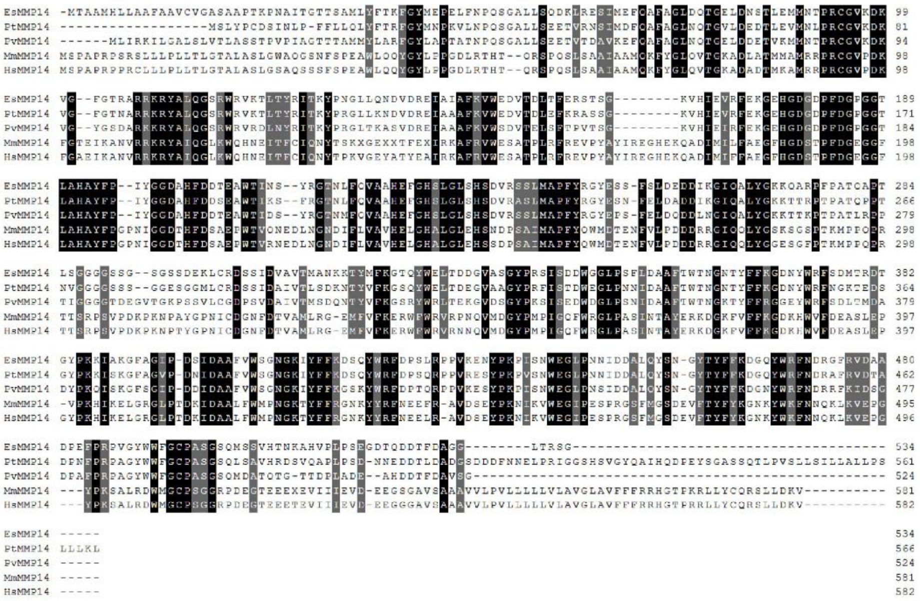

B

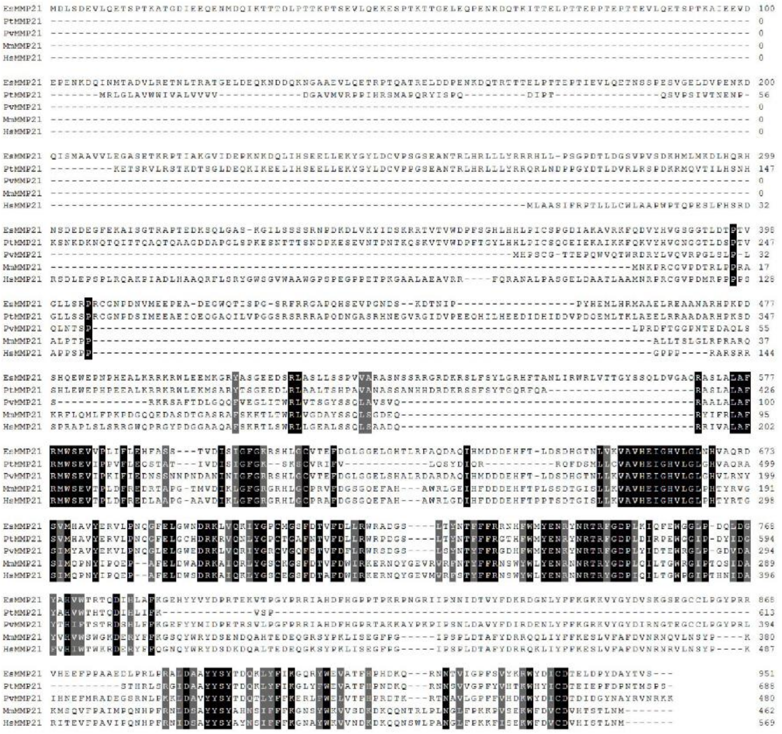

Supplementary Figure S3. The multiple sequence alignment of es-MMP14 (**A**) and es-MMP21 (**B**) proteins.

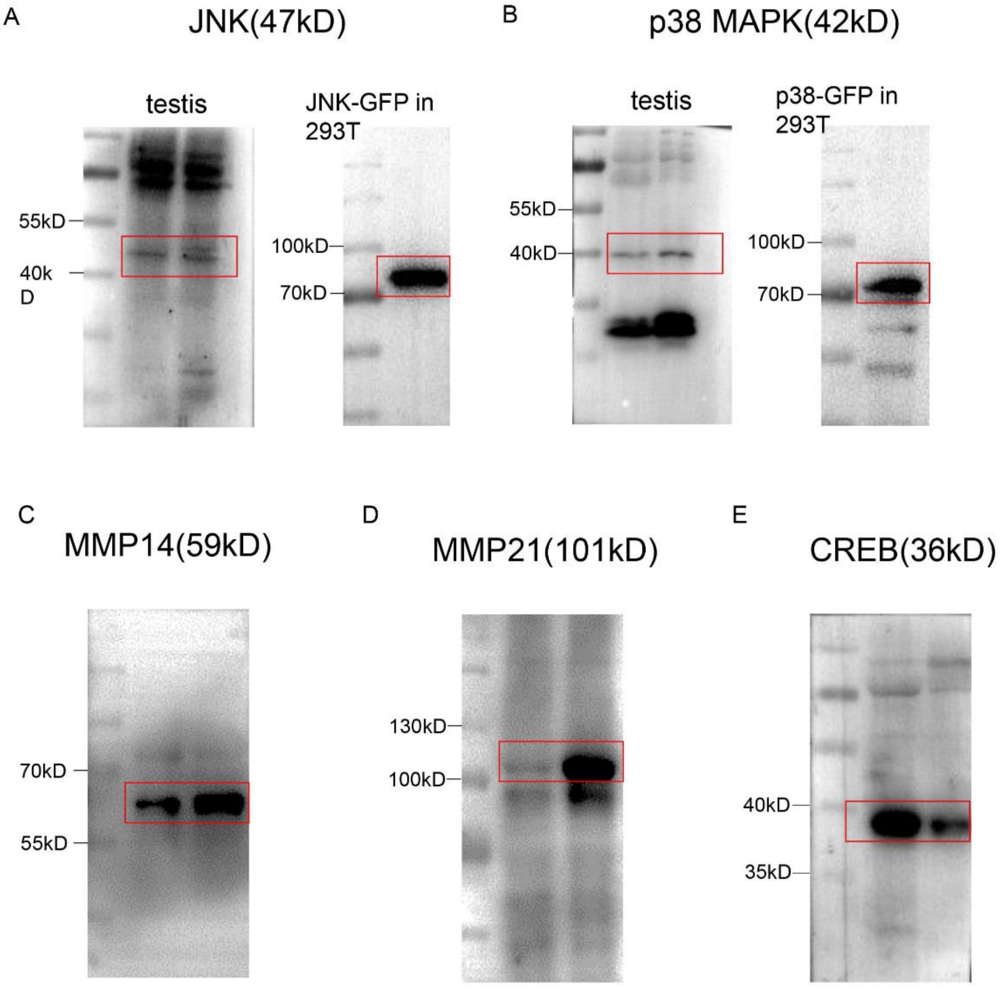

Supplementary Figure S4. The verification of specificity of es-JNK, es-p38 MAPKA, es-MMP14, es-MMP21 and es-CREB antibodies in *E. sinensis* and HEK293t cell line. (**A**,**B**) We verified the antibodies of es-JNK an es-p38 MAPK in *E. sinensis* testis total protein and HEK293T which overexpressed with es-JNK or es-p38 MAPK. There were obvious bands in the western blotting results. (**C**–**E**) We verified the antibodies of es-MMP14, es-MMP21 and es-CREB in *E. sinensis* testis total protein, we found the single target band. We used the primary antibodies with 1:1000 dilution and 1:5000 for the second antibody.
